# Intronic L1 Retrotransposons and Nested Genes Cause Transcriptional Interference by Inducing Intron Retention, Exonization and Cryptic Polyadenylation

**DOI:** 10.1371/journal.pone.0026099

**Published:** 2011-10-13

**Authors:** Kristel Kaer, Jelena Branovets, Anni Hallikma, Pilvi Nigumann, Mart Speek

**Affiliations:** Department of Gene Technology, Tallinn University of Technology, Tallinn, Estonia; Keio University, Japan

## Abstract

**Background:**

Transcriptional interference has been recently recognized as an unexpectedly complex and mostly negative regulation of genes. Despite a relatively few studies that emerged in recent years, it has been demonstrated that a readthrough transcription derived from one gene can influence the transcription of another overlapping or nested gene. However, the molecular effects resulting from this interaction are largely unknown.

**Methodology/Principal Findings:**

Using *in silico* chromosome walking, we searched for prematurely terminated transcripts bearing signatures of intron retention or exonization of intronic sequence at their 3′ ends upstream to human L1 retrotransposons, protein-coding and noncoding nested genes. We demonstrate that transcriptional interference induced by intronic L1s (or other repeated DNAs) and nested genes could be characterized by intron retention, forced exonization and cryptic polyadenylation. These molecular effects were revealed from the analysis of endogenous transcripts derived from different cell lines and tissues and confirmed by the expression of three minigenes in cell culture. While intron retention and exonization were comparably observed in introns upstream to L1s, forced exonization was preferentially detected in nested genes. Transcriptional interference induced by L1 or nested genes was dependent on the presence or absence of cryptic splice sites, affected the inclusion or exclusion of the upstream exon and the use of cryptic polyadenylation signals.

**Conclusions/Significance:**

Our results suggest that transcriptional interference induced by intronic L1s and nested genes could influence the transcription of the large number of genes in normal as well as in tumor tissues. Therefore, this type of interference could have a major impact on the regulation of the host gene expression.

## Introduction

Genes in mammalian chromosomes are distributed between gene-poor and gene-rich territories. While individual genes in gene-poor regions can be regulated independently without interference from others, genes in gene-rich regions can be regulated via complex network of positive and negative interactions. Gene-gene interactions, occurring at transcriptional level, have been recently recognized as a widespread and unexpectedly complex process, termed transcriptional interference (TI) [1], [2]. According to [1], TI is defined as suppressive influence of one transcriptional process or an RNA polymerase II (Pol II) complex on a second transcriptional process. Most of the TI occurs between two genes and depends on their transcriptional orientation and/or arrangements. Genes with convergent promoters generate overlapping transcripts from opposite strands. Tandemly oriented genes produce frequently overlapping transcripts from the same strand. Divergently oriented genes, an abundant class of genes, generate transcripts from opposite strands using bidirectional promoters [3]. Depending on the orientation of genes, several mechanisms of TI have been proposed. These include promoter competition in divergent, ”sitting duck” in tandem or convergent and collision in convergent arrangements of genes (reviewed in [1]). In all cases TI occurring between genes can lead to premature termination of initiation or elongation of either one, or the other, or both Pol II complexes. Initial TI studies were carried out mostly with artificial constructs, i.e. with genetically engineered transcription units [4], [5], [6], [7], [8], [9]. However, recently several naturally occurring examples of TI were also studied in bacteria [10], [11], [12], yeast [13], [14], fly [15], [16] and mammals [17], [18], [19], [20].

One interesting example, a gene within a gene, or so called nested gene, has been in the focus in several recent studies (reviewed in [21], [22]). It has been demonstrated that about 63 % of the nested human genes are transcribed from the opposite strand. And about 41 % of the nested genes are single exon genes [23]. Many nested genes are expressed in specific tissues and some of them show negative correlation with the host gene expression. In another study [24], functional significance of nested genes has been questioned. Since neither positive nor negative correlation in the expression of 109 human and 752 fly nested genes was observed, their results supported a neutral theory of coevolution.

Similarly to naturally occurring nested genes, a Russian doll-type arrangement of genes could be generated as a result of mobile element (for example, L1 retrotransposon) insertion into an intron of the host gene. Human genome contains 80–100 retrotranspositionally competent L1s [25] and about 7000 full-length L1s [26], which have lost their transposition capacity because of point mutations interrupting their open reading frames (ORFs). About half of these L1s are located in introns and the rest are scattered over intergenic regions [27]. It is not known how many of them have retained the transcriptional potential, although hundreds, or perhaps even thousands of L1-derived transcripts, could contribute to the human transcriptome [28], [29].

Transcriptional activity of human L1 is determined by convergently arranged sense (SP) and antisense (ASP) promoters, located in its 5′ untranslated region (5′ UTR) in positions +1 to +101 and +400 to +600, respectively [30], [31]. SP is required for retrotransposition and its actvity depends on Yin Yang-1 (YY1) (positions +13 to +21) [32], SOX family members (positions +472 to +477 and +572 to +577) [33] and RUNX3 (positions +83 to +101) transcription factors (TF) [34]. L1 ASP drives transcription of nearby genes in opposite direction yielding chimeric transcripts [35], [36] and so far only one binding site of RUNX3 (positions −526 to −508 opposite strand), required for L1 ASP activity, has been determined [34]. It has been shown that intronic L1s, containing 19 canonical and noncanonical polyadenylation (polyA) signals in its sense strand (ORF1 and ORF2), can interfere with transcriptional elongation and cause premature polyadenylation [37], [38]. However, the potential TI effects of L1 SP and ASP, whether arranged tandemly or convergently with respect to the host gene transcription, have not been analyzed.

Here we studied the effect of L1 SP- and ASP-induced TI on human genes. We also extended our studies to protein-coding and noncoding (nc) nested genes, i.e. a category of genes, for which only a limited information about transcriptional regulation is available. Our results show that intron retention, forced exonization of intronic sequence and cryptic polyadenylation at the 3′ ends of prematurely terminated transcripts are the major effects induced by intronic L1s or nested genes. These effects were deduced from aberrant endogenous transcripts expressed in different human cell lines and tissues and confirmed in minigene transfection experiments.

## Results

### Prediction of transcriptional interference (TI) induced by human L1 retrotransposons

**Figure 1:**
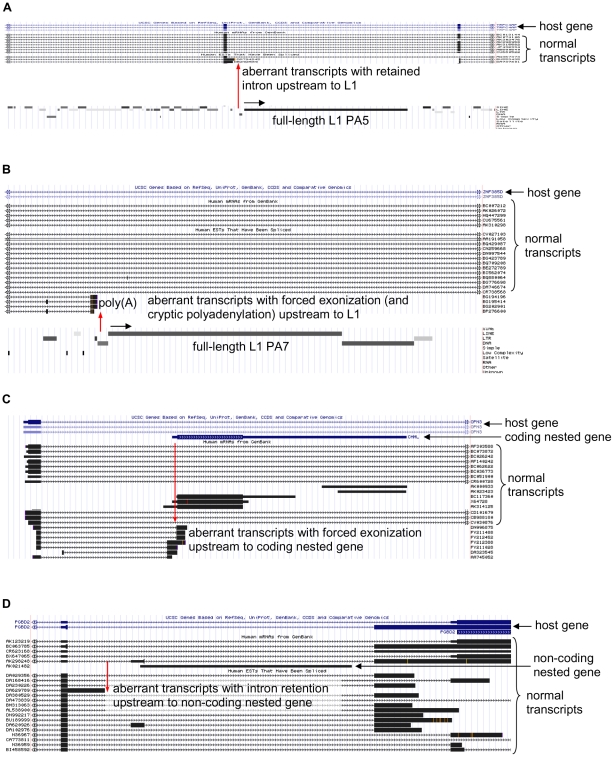
Snapshot of L1- and nested gene-induced TI from UCSC Genome Browser [**39**]. Schematic representation of (A) intron retention in *TRPC4AP* (ESTs: DA794245, DB292589) upstream to full-length L1 PA5 element, (B) forced exonization and cryptic polyadenylation in *ZNF385D* (ESTs: BG194196, BG195414, BG202901, BP276600) upstream to full-length L1 PA7 element, (C) exonization in *OPN3* (ESTs: DN996875, DA323545, AA745052 etc) upstream to single exon coding nested gene *CHML* and (D) intron retention in *PGBD2* (EST: DA629789) upstream to putative nc nested gene (EST: AK021482). Arrows above L1 represent the direction of transcription. Red arrows mark the 3′ ends of aberrant transcripts. Although the cryptic polyadenylation is shown only in panel B, it was variably found in either exonization or intron retention products (Figure 8).

Using *in silico* chromosome walking with UCSC Genome Browser [39], we carried out a genome-wide search for prematurely terminated transcripts bearing signatures of intron retention, forced exonization and cryptic polyadenylation at their 3′ ends upstream to the human intronic L1 retrotransposons. Representative examples of two genes containing aberrant transcripts showing intron retention and exonization are shown in **Figure 1** **A and B**. The term intron retention, used here, refers to the unspliced intron, which is retained in the 3′ end of spliced exons most likely due to the premature termination of Pol II possibly induced by L1. Similarly, a different molecular effect, forced exonization of the intronic region upstream to L1, describes TI effect, which may be caused by either transcriptional activity of L1 or TFs bound to L1. Exonization depends on the presence of upstream cryptic acceptor splice site.

Our genome-wide search revealed 50 intronic L1s in 49 genes which had a tandem arrangement with respect to the host gene transcription and possibly caused intron retention, forced exonization and cryptic polyadenylation in the upstream region (**Table S1**). Of the L1s analyzed, intron retention was observed in 22 genes (45 %) and forced exonization in 19 genes (39 %). Both, intron retention and forced exonization were observed in 8 genes (16 %). Cryptic polyadenylation was detected in 13 genes (27 %). Of these genes, 5 showed intron retention, 3 exonization and 5 had both effects.

In order to determine if the L1s analyzed are the only potential inducers of TI, we searched for additional aberrant expressed sequence tags (ESTs) in our dataset of 49 genes (**Table S2**). None of this type of ESTs were found in 17 genes (35 %). However, for the remaining 31 genes (63 %) we detected additional potential TI effects in a single intron of 15 genes and in two or more introns in 16 genes. For these 31 genes intron retention was observed in 10 genes (32.5 %), exonization in 11 genes (35 %) and both effects in 10 genes (32.5 %). The occurrence of aberrant ESTs in these cases may be explained by the presence of either repeated DNA (Alu, MIR, SVA, etc) or a putative nested gene, which could influence the transcription across the intron (see also below). Indeed, their direct influence, i. e. intron retention, exonization, termination or polyadenylation within the repeated DNA or nested gene was detected in 18 genes (58 %). Of 49 genes, only in one case (KIAA0586) we were unable to explain the intron retention in mRNA AK302718 (marked with ? in **Table S2**). From these data we conclude that in addition to L1, other repeated DNAs as well as nested genes, located in introns of host genes, could possibly cause TI effects in these genes.

### Prediction of TI for protein-coding nested genes

Since TI may occur between any pair of tandemly arranged genes, we predicted that nested genes might also interfere with transcription of host genes. Previously, Yu *et al*. [23] analyzed the human genome (UCSC Genome Browser Oct 2004, NCBI Build 34) and found 373 reliably annotated nested genes. Of these genes, 158 represented protein-coding genes, 212 pseudogenes and 3 snoRNA genes. Of the 158 protein-coding genes, only about one third (53 genes) were tandemly arranged. We reevaluated their data and found that 31 of them were probably individual and tandemly arranged genes. The remaining 22 nested genes were most likely alternatively spliced variants (5′ or 3′ exons) derived from the host genes and thus, were not truly nested. We searched for potential TI effects in their group of 31 nested genes and discovered that 7 of them could interfere with the host gene transcription by causing exonization and intron retention in their upstream region (**Table 1**). Using a more recent assembly (Mar. 2006), we also carried out an independent search for nested genes possibly involved in TI in chromosome 18, for which no nested genes were found earlier [23]. Our search revealed two new protein-coding nested genes (**Table 1**). This result suggests that the actual number of nested genes, and those involved in TI, may be higher.

**Table 1:** Prediction of TI between host and protein-coding nested genes.

No	UCSC Genome Browser	Host gene	Number of exons	Nested gene	Location in intron	TI EST (etc >3 ESTs)	Expession	Effects
1	chr1:52,258,391–52,293,635	*TXNDC12*	7	*KTI12*	2	DA735586	teratocarcinoma cell line	exonization ∼0.3 kb upstream, inclusion
						DA277685	corpus callosum	exonization ∼0.3 kb upstream, inclusion
						DA719333	teratocarcinoma cell line	exonization ∼0.3 kb upstream, inclusion
2	chr1:178,080,576–178,113,645	*TOR1AIP2*	6	*IFRG15*	2	BX324877	placenta	exonization ∼0.7 kb upstream, inclusion
						BQ214560	melanotic melanoma	exonization ∼0.7 kb upstream, inclusion
						BX110121 etc	placenta	exonization ∼0.7 kb upstream
3	chr1:239,823,075–239,870,324	*OPN3*	4	*CHML*	1	AA745052	breast tumor	exonization ∼0.1 kb upstream
						DA323545	hippocampus	exonization ∼0.1 kb upstream, inclusion
						DN996875	breast cancer	exonization ∼0.1 kb downstream, inclusion
4	chr3:73,128,809–73,197,701	*PPP4R2*	9	*FLJ10213*	5	BM921339	pooled brain, lung, testis	intron retention ∼0.6 kb upstream
5	chr5:60,205,416–60,276,662	*ERCC8*	13	*FLJ12595*	10	BG032140	mammary adenocarcinoma cell line	exonization ∼0.1 kb upstream, inclusion
6	chr7:127,079,438–127,519,895	*SND1*	24	*C7orf54*	16	BF966905	hippocampus	exonization 37 b upstream, inclusion
7	chr7:149,651,229–149,666,178	*LRRC61*	3	*C7orf29*	2	BQ949548	lung large cell carcinoma	exonization ∼1.2 kb upstream
						CT003407	T-Lymphocytes	exonization ∼1.2 kb upstream
						DB239558 etc	trachea	exonization ∼1.2 kb upstream
8	chr18:10,444,625–10,478,698	*APCDD1*	5	*LOC100130468*	3	BU541433	prostate carcinoma cell line	exonization 37 b upstream
9	chr18:74,930,385–75,239,271	*ATP9B*	29	*LOC653054*	6	CD654775	embryonic stem cells	intron retention ∼0.6 kb upstream

### TI may also be induced by nested noncoding RNA genes

Despite the fact that biological function of the vast majority of nc RNA transcripts, an abundant class of RNAs (over 55 00) [40], [41], [42], is largely unknown, our results of L1 and protein-coding nested genes predicted that nc RNA genes might also be involved in the induction of TI. Therefore, we decided to undertake a preliminary analysis of TI effects induced by putative protein-noncoding nested genes. For this purpose, we carried out *in silico* chromosomal walking using human chromosomes 1, 3 and 13 (randomly chosen, approx. 1/5 of the genome) and selected putative single exon nested genes, for which at least one TI-predicting EST was found. Representative examples of one protein-coding and nc nested genes are shown in **Figure 1** **C and D**. Our search revealed 104 nested genes, of which 94 (90 %) were potential nc genes and 10 (10 %) were protein-coding (**Table S3**). Of these protein-coding genes, 4 were detected earlier (**Table 1**). All nested genes analyzed were located within introns of 74 host genes: 51 of them contained one and 23 contained two or three nested genes. For these nested genes, TI analysis revealed exonization in 68 cases (65 %), intron retention in 34 cases (33 %) and both effects in 2 cases (2 %). Cryptic polyadenylation was detected in 19 genes (18 %). Of these genes, 16 showed exonization, 2 intron retention and 1 had both effects. Comparison with L1 data (**Table S1**) also revealed that in the case of nested genes exonization was more prevalent (about 2-fold) than in the case of intronic L1s (see above).

To test whether the potential TI effect in host gene expression is specific only to the intron, wherein the nested gene is located, we searched for aberrant ESTs over the entire length of each host gene (**Table S4**). Of the 74 host genes analyzed, 26 (35 %) showed no additional aberrant ESTs demonstrating that in these cases the nested gene was the only potential TI inducer. The next large group of 43 host genes (58 %) showed variable number of aberrant ESTs in different introns; intron retention in 15 genes, exonization in 14 and both effects in 16 host genes. Their presence in these introns may be explained by the TI effect induced by repeated DNA (Alu, MIR, L1, L2, HERV, etc) similar to that described above for L1-containing genes. Consistent with this explanation, their direct influence, i. e. intron retention, exonization, premature termination or polyadenylation within the repeated DNA was detected in 17 cases (40 %). Finally, we also detected 5 host genes (7 % of total 74 genes), which expression gave rise to aberrant ESTs (exonization in 1 case and intron retention in 4 cases) in other introns (marked with ? in **Table S4**). Their presence in these few cases remained unknown. Taken together, these data show that in addition to intronic nested genes, pseudogenes or repeated DNAs can potentially induce the major TI effects compared to minor effects (43 vs 5), caused by other unknown factors (e.g., transcriptional elongation constraints).

### TI between nested genes may be detected from endogenous transcripts derived from different human tissues

**Figure 2:**
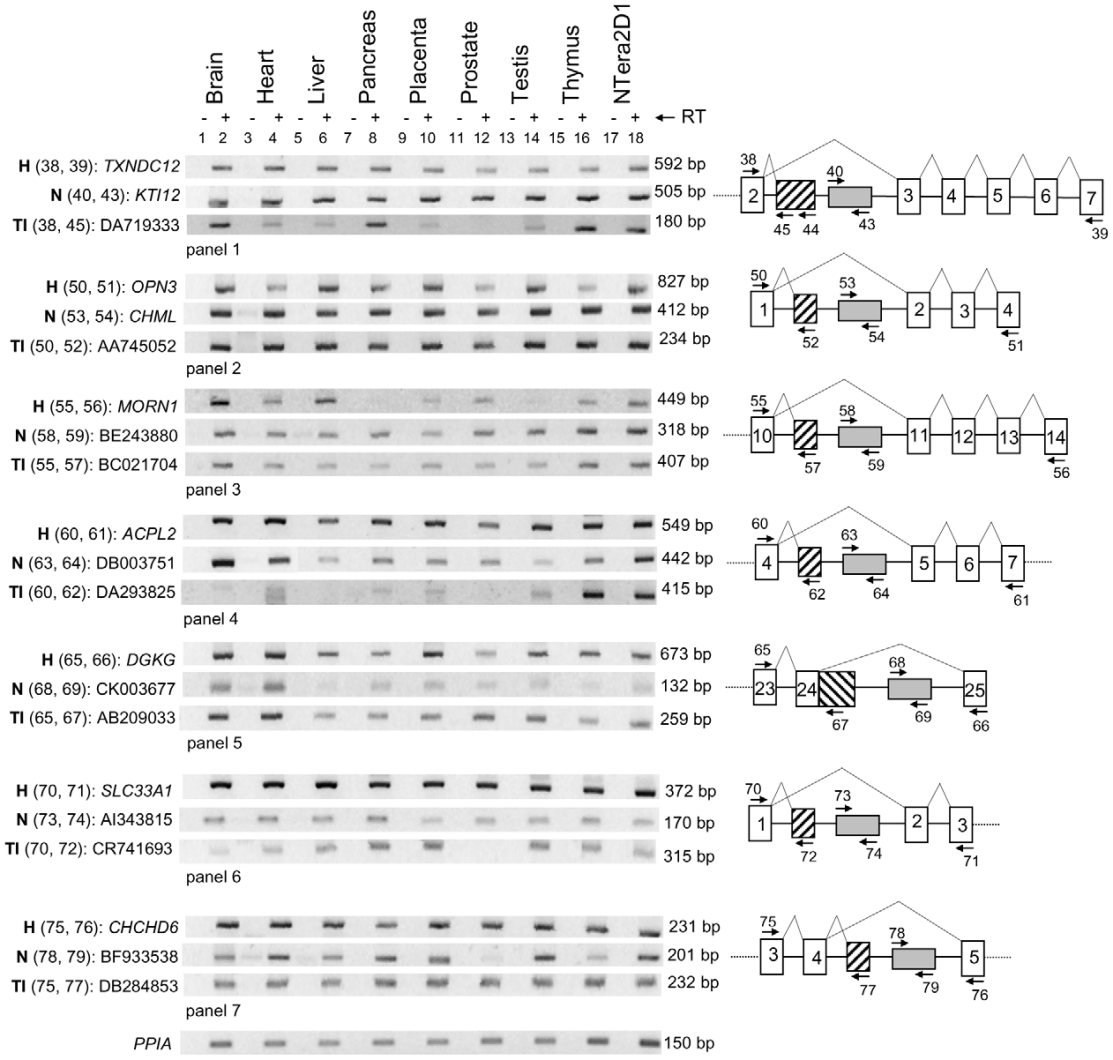
TI effects induced by protein-coding and putative nc nested genes in different human tissues. Analysis of the transcripts of five nc (panels 3–7) and two protein-coding nested genes (panel 1 and 2). Endogeneous host gene (H), nested gene (N) and host gene transcripts showing exonization and intron retention upstream to the nested genes (TI) were detected by RT-PCR. Intron-containing transcripts in *TXNDC12* were further amplified by nested PCR. Names of the host-nested genes and TI transcripts are indicated on the left and length of the products on the right side of panels, respectively. Corresponding exon-intron structures (not in scale) for nested genes are shown on the right of panels. Nested genes are shown with grey boxes, exonization of intronic sequence upstream to nested gene is shown with hatched box with upward diagonals and intron retention with downward diagonals. Exons are shown with white boxes, introns with lines and splicing with diagonal lines. Primers used for each (H, N and TI) amplification are shown in parenthesis on the left side and their numbers refer to the corresponding product shown on the right side of each panel. Their numbers correspond to sequences listed in Table S5. Plus and minus signs represent experiments in the presence or absence of reverse transcriptase (RT). *PPIA* – positive control; NTera2D1 – human teratocarcinoma cell line.

As a first step toward understanding the potential TI effect induced by nested genes, 7 nested genes (5 nc and 2 protein-coding) were randomly chosen (**Table S3**). For these genes, we tested the presence of normal transcripts for host and nested and aberrant transcripts for host genes in various human tissues. Widespread transcription of host and nested genes was detected in all tissues analyzed, which in most cases matched the potential TI effect induced by nested gene (**Figure 2** panels 1, 2, 4, 5 and 6). The lack of aberrant transcripts in some tissues where the host and nested genes were both transcribed suggests that their activity may be restricted to different cell populations (panel 1, lane 12; panel 4, lanes 6 and 12; panel 6, lane 12). The absence of host gene transcription in some tissues where nested gene and aberrant transcripts were expressed may be due to alternative splicing (panel 3, lanes 8 and 14). Also, in one case the nested gene was not transcribed, but the presence of aberrant transcript (panel 7, lane 12) suggested that binding of TFs to nested gene, could be responsible for the observed TI effect. Despite these exceptions, our results show that TI between protein-coding and nc nested genes may be widespread and in some cases restricted to certain human tissues.

### Selection of three genes (*ABCA9*, *NCAM1* and *TXNDC12*) for detailed mapping and further analysis of potential TI effects in minigene transfection experiments

**Figure 3:**
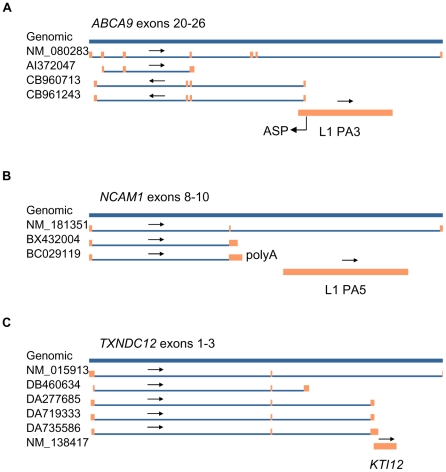
Mapping of ESTs used for the prediction of TI in three human genes. (A) *ABCA9* mRNA - NM_080283, ESTs: AI372047, CB960713, CB961243 and L1 PA3 were mapped to its genomic structure containing exons 20 to 26. L1 ASP drives transcription from the opposite strand and produces ESTs CB960713 and CB961243. Intron retention to exon 23 is shown in EST AI372047. (B) *NCAM1* mRNA - NM_181351, ESTs: BX432004, BC029119 and L1 PA5 were mapped to its genomic structure containing exons 8 to10. Intron retention to exon 9 is observed in two ESTs (BX432004 and BC029119) one of which is polyadenylated. (C) *TXNDC12* mRNA - NM_015913, ESTs: DB460634, DA277685, DA719333, DA735586 and *KTI12* mRNA - NM_138417 were mapped to its genomic structure containing exons 1 to 3. Exonization upstream to *KTI12* was observed in four ESTs (DB460634, DA277685, DA719333 and DA735586). Genomic DNA is marked with blue line on top. Orange boxes refer to exons, blue lines to introns and arrows indicate the direction of transcription. L1 and *KTI12* are shown with orange thick lines. Detailed mapping was carried out with Spidey [69].

To study potential TI effects in more detail, three host genes *ABCA9*, *NCAM1* and *TXNDC12* were chosen for further analysis. *ABCA9* (ATP-binding cassette, subfamily A, member 9) is located in chromosome 17q24.2, consists of 39 exons and encodes a transporter protein which transports different molecules through extra- and intracellular membranes [43]. In *ABCA9,* a full-length L1 PA3 is located in intron 25 and it was previously shown that this L1 has a functional ASP [36]. This promoter is able to produce a spliced antisense RNA (ESTs CB960713 and CB961243) containing a 101 nucleotide (nt)-long region complementary to *ABCA9* exon 23 (**Figure 3A**). Although L1 PA3 is located in intron 25, we predicted that transcription from L1 ASP may interfere with the transcription and splicing of the *ABCA9* by causing intron retention in exon 23, as exemplified by EST AI372047.

*NCAM1* (Neural cell adhesion molecule 1) is located in chromosome 11q23.1, consists of 19 exons and encodes a protein involved in neural cell adhesion [44]. A full-length L1 PA5 is located in intron 9 of *NCAM1* (**Table S1**) and may interfere with its transcription by causing intron retention and cryptic polyadenylation about 2 kb upstream to L1 (**Figure 3B**). This prediction is based on two ESTs: BX432004 and BC029119.

*TXNDC12* (thioredoxin domain containing 12) is located in chromosome 1p32.3, consists of 7 exons and encodes a thioredoxin domain-containing protein involved in redox regulation and defense against oxidative stress [45]. Second intron of *TXNDC12* contains a tandemly oriented single exon gene (*KTI12*), which encodes a chromatin associated protein of unknown function (**Table 1**) [46]. This gene was selected because of its small size (1.7 kb) and its potential TI effect was restricted to some tissues (**Figure 2**). Our data predict that nested *KTI12* interferes with *TXNDC12* transcription by forcing exonization 251 nt upstream to its transcriptional start site. This effect may be deduced from 3 different ESTs: DA277685, DA719333 and DA735586 (**Figure 3C**). In addition, a single EST: DB460634 showed exonization about 5 kb upstream to *KTI12*.

### L1-induced transcription interferes strongly with the SV40 transcription and causes intron retention and exonization in ABCA minigene

**Figure 4:**
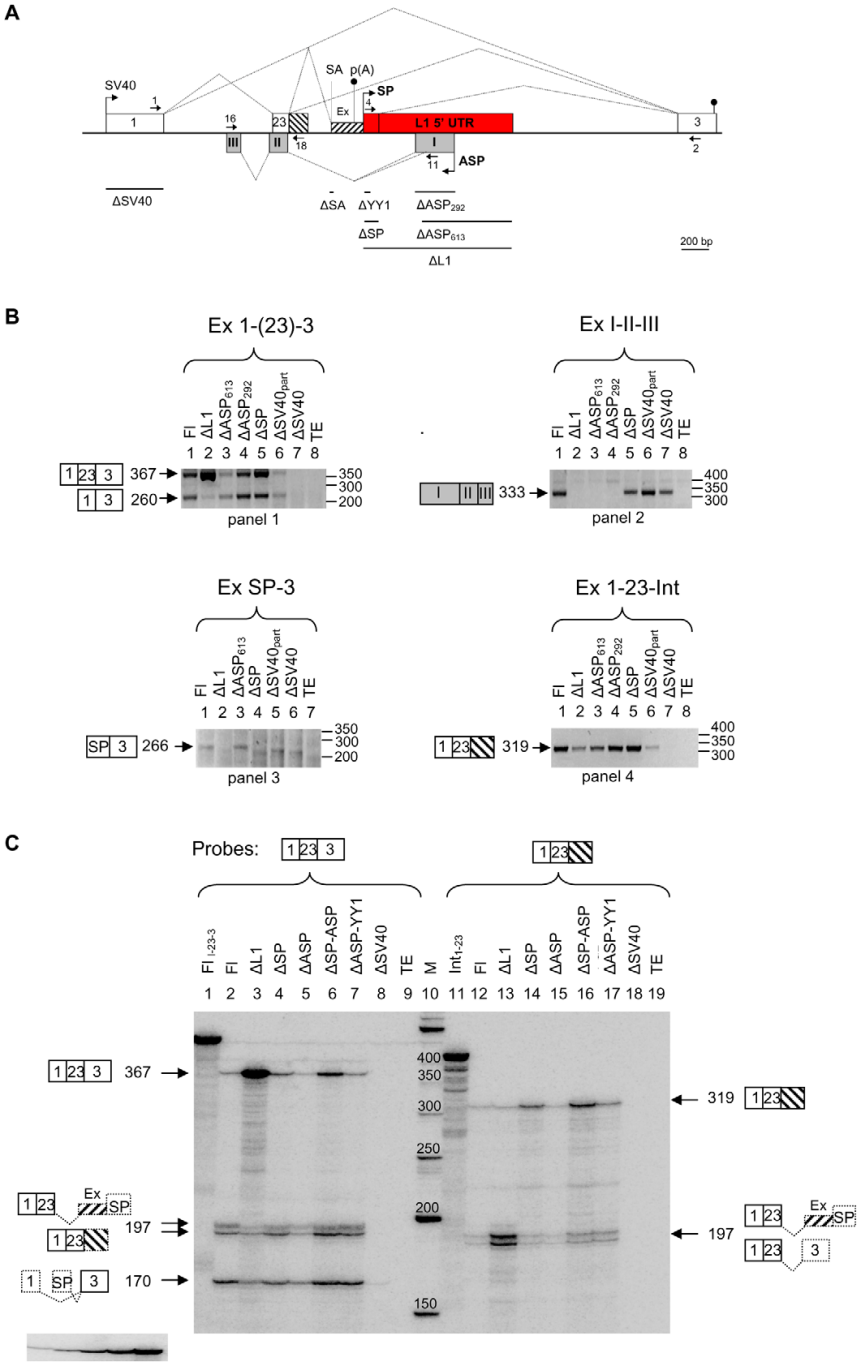
L1-induced transcription decreases SV40 transcription and causes intron retention in ABCA minigene. (A) Schematic representation of the ABCA minigene and its deletion constructs used in transfection experiments. Splicing of exons observed in different transcripts is shown by diagonal lines. Deletions made in ABCA Fl construct are marked with lines below the scheme. Intron retention to exon 23 is shown by hatched box with downward diagonals. Exonization of intronic sequence upstream to L1 5′ UTR is shown with hatched box with upward diagonals and marked with Ex. In this exon cryptic acceptor splice site (SA) and polyA signal (p(A)) are marked. Additional SV40 polyA signal (marked with lollipop) is located in exon 3. L1 ASP drives transcription from the opposite strand and produces transcript containing exons I, II and III (grey boxes). Its structure is shown on panel A bottom strand. Primers used in RT-PCR are shown with small arrows and their sequences are listed in Table S5. (B) RT-PCR of various transcripts expressed from minigene constructs. Transcripts, containing exons or introns (shown on top of each panel) derived from SV40 promoter and L1 SP/ASP were reverse transcribed and amplified by PCR. The obtained products are shown on the left of each panel and their labeling is according to the scheme A. Size markers (in bp) are shown on the right of each panel. (C) Quantitative detection of various minigene transcripts by RPA. Riboprobes Fl_1-23-3_ and Int_1-23_ (lanes1 and 11) are schematically shown above each panel. Fl and different deletion (Δ) constructs (ΔASP = ΔASP_292_) used in transfection experiments are shown on top of each lane. Protected transcripts (marked with arrows) are schematically shown by boxes and their sizes are given in nucleotides. Dashed lines/boxes show the remaining exon(s) not protected by the riboprobe used. TE – transfection simulated with buffer. Autoradiogram clip on the bottom left shows a 2-fold serial dilution of the probe used in quantitation of transcripts.

Based on the results of our bioinformatic study (**Figure 3**), we decided to use minigene constructs for the analysis of potential TI effects. A so-called full-length (Fl) ABCA minigene was constructed by insertion of a 1036 bp genomic fragment containing intron 22, exon 23 and intron 23 of *ABCA9* and a 990 bp L1 5′ UTR (#11AS) [31] into exon trapping vector pSPL3 containing SV40 promoter (**Figure 4A**) [47]. Subsequently, from this construct various deletions were made (for details see Materials and Methods). All constructs were transfected into human teratocarcinoma cells supporting SV40 and L1 SP/ASP transcription. In the first series of experiments, qualitative RT-PCR analysis was carried out (**Figure 4B**). Two SV40 transcripts were detected: a 367 nt transcript containing exons 1, 23 and 3, and a 260 nt transcript containing exons 1 and 3, skipping exon 23 (**Figure 4B** panel 1). Increased SV40 transcription (transcript 1-23-3) was observed after deletion of the entire L1 5′ UTR, suggesting that L1 SP/ASP could strongly interfere with SV40 transcription (cf. lanes 1 and 2). A small variation in the amount of alternative splice variant was observed depending on the deletion used. SV40 transcription was dependent on the intactness of the promoter (cf. lanes 6 and 7). For L1 ASP-driven transcription a spliced transcript of the expected size 333 nt (exons I-II-III) was observed for all L1 ASP-containing constructs indicating that L1 ASP was active (**Figure 4B** panel 2**,** cf. lanes 1 and 5–7). Consistent with this result, a small (ΔASP_292_) or a large (ΔASP_613_) deletion encompassing L1 ASP region [31], containing RUNX3 binding site [34], eliminated its activity. Also, an L1 SP-containing transcript (SP spliced to exon 3) of the expected size (266 nt) was detected in the background of endogenous L1 transcripts (**Figure 4B** panel 3**,** lanes 1 and 3). This transcript was absent in negative control (ΔSP, lane 4) and in ΔSV40 experiments (lanes 5–6), suggesting that SP activity was dependent from the SV40 promoter activity. Finally, SV40 spliced transcripts of the expected size (319 bp) with retained intron 23 were detected for all constructs, except for ΔSV40 (**Figure 4B** panel 4). Variation in the intensity of these products correlated with the presence or absence of L1 5′ UTR, or part of it, suggesting that it may interfere with the intron retention (cf. lanes 1–3). All these RT-PCR experiments (including those described below) were first used to diagnose (qualitatively) the potential TI effects and thereafter the products were identified by restriction analysis, cloned and sequenced.

To study the potential of L1-induced TI in more detail, we chose the RNase protection assay (RPA). Since this method is based on solution hybridization and allows quantitative and simultaneous detection of different (un)spliced transcripts, contributions of each promoter or transcription unit can be determined. **Figure 4C** shows the detection of different SV40 transcripts by using Fl and various minigene deletion constructs. The following observations can be made from this experiment. Deletion of the entire L1 transcription unit increased SV40 transcription (and splicing) about 8-fold suggesting that L1 5′ UTR interferes strongly with the elongating SV40 Pol II (cf. lanes 2 and 3). This result also shows that the presence of L1 5′ UTR in intron 23 makes SV40 transcription and splicing about 8 times less efficient and thus may affect intron 23 retention to spliced exons 1 and 23. Indeed, the same deletion also increased the ratio of Fl to intron-retained transcripts about 8-fold (cf. lanes 12 and 13). Deletion of the L1 SP, containing YY1 and RUNX3 binding sites [32], [34], increased SV40 transcription about 2-fold, suggesting that tandemly located L1 SP can act as a roadblock or a sitting duck (cf. lanes 2 and 4) [1]. Surprisingly, however, this deletion also increased intron retention about 2-fold (cf. lanes 12 and 14), suggesting that L1 SP could affect processing of SV40 transcripts and exonization in intron 23 and SP region most likely by providing donor splice site. It is possible that the presence of L1 SP splicing donor site at position 96 in a sequence ATCTGAGgtaccggg [48] is somehow beneficial for SV40 Pol II to guarantee processive transcription coupled with splicing across the exons 23, ExSP and 3 (**Figure 4A**), whereas the absence of it (in ΔSP) may force Pol II to slow down or dissociate from the template by producing intron-containing transcripts (see also below). Deletion of the L1 ASP alone did not affect the level of SV40 transcription and splicing when compared with Fl construct (**Figure 4C** lanes 2 and 5). However, deletion of both L1 ASP and SP caused about 3-fold increase in the transcription, and to a lesser extent increased the intron retention, compared to L1 SP deletion alone (cf. lanes 4 and 6, and 14 and 16), suggesting that combination of L1 SP and ASP could synergistically contribute to the TI. Consistent with that conclusion, deletion of the entire L1 5′ UTR sequence showed a far greater effect than the combined deletion of L1 SP and ASP (cf. lanes 3 and 6, and 13 and 16) suggesting that other regions in L1 5′ UTR also contributed to the TI. Deletion of the YY1 binding site of SP (positions +13 to +21 in L1 5′ UTR) [32] combined with the L1 ASP deletion increased slightly both transcription and intron retention (cf. lanes 5 and 7, and 15 and 17) suggesting that this region somehow contributes to the TI.

Previous studies have suggested that SOX family TFs can modulate L1 SP activity [33] and SOX2 may be involved in the transcription and retrotransposition of L1 [49]. In our minigene experiments, mutations of two SOX binding sites in positions +472 to +477 and +572 to +577 [33] did not affect the ratio of Fl transcripts to intron-containing transcripts, suggesting that SOX factors alone may not be responsible for the observed effect (**Figure S1A,** cf. lanes 3 and 6–8, and 13 and 16–18). We also tested potential TI effect of L1 PA3 derived from the *ABCA9* intron 25 and compared it to the #11AS in ABCA Fl and retrotranspositionally active L1 RP [50]. Both, L1 PA3 and RP 5′ UTR were about 2–4 fold less effective in inducing TI than L1 #11AS [31] (**Figure S1A,** cf lanes 3, 9 and 10 or 13, 19 and 20). Full-length L1 PA3 and RP reduced significantly SV40 transcription, suggesting that these elements are rather difficult to transcribe (**Figure S1B,** lanes 2–4 and 9–11). Full-length L1 RP and ORFs (to a lesser extent) in sense orientation had much greater effect in TI than antisense orientation (lanes 2–3 and 5–6), consistent with previous studies [37], [38], [48], [51]. All these data suggest that tandemly arranged L1 could affect transcriptional elongation through its SP/ASP transcriptional activity and/or TF binding, while ORFs could nonspecifically inhibit transcriptional elongation of the host gene.

**Figure 5:**
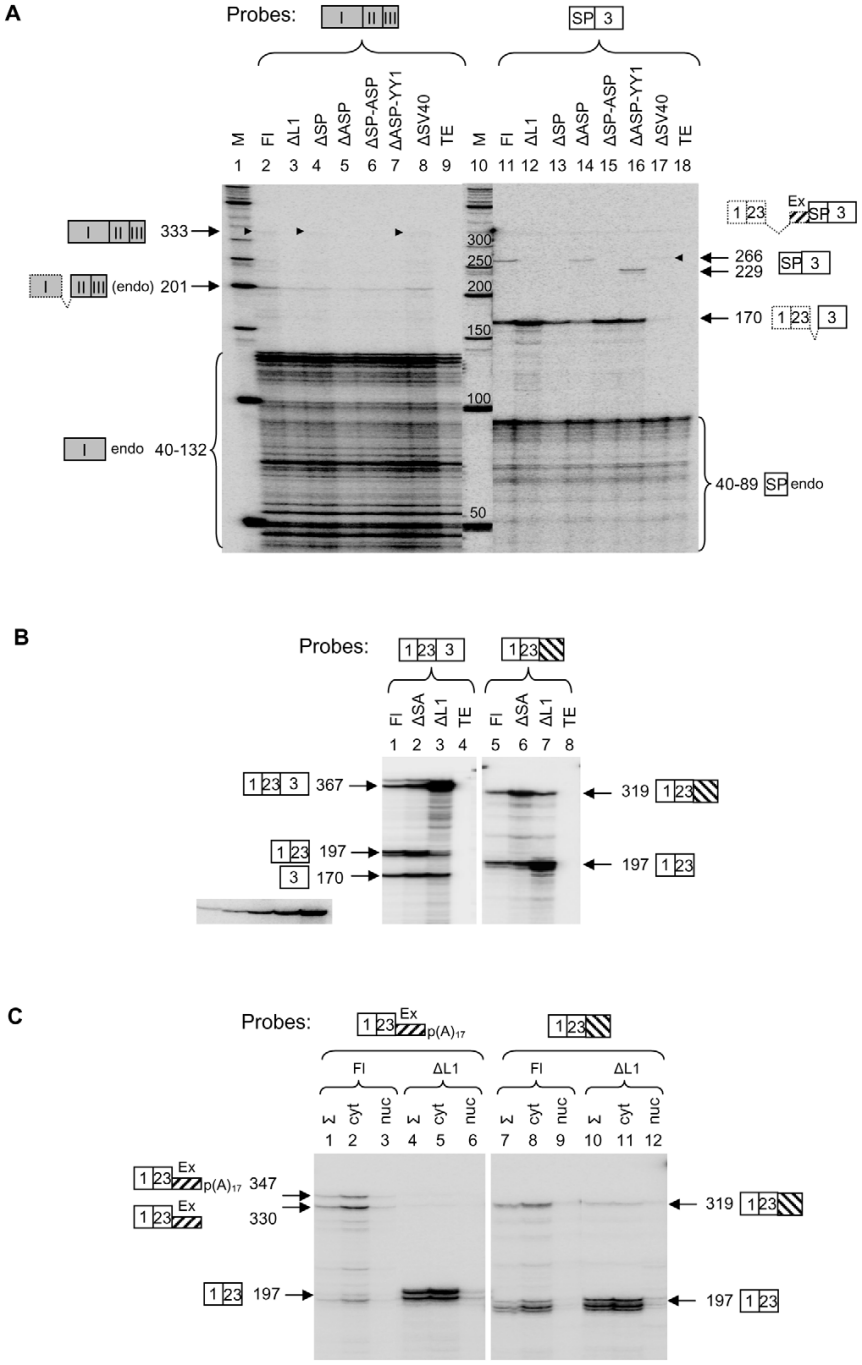
TI effects of L1 SP and ASP, cryptic splice site and polyA signals in ABCA minigene. (A) Analysis of the L1 ASP- and SP-driven transcripts expressed from various minigene constructs (ΔASP = ΔASP_292_). Note the large number of endogenous transcripts (endo) with sizes 40–132 nt and 40–89 nt hybridizing to exon I and SP, respectively. (B) Deletion of acceptor splice site increases intron retention. Transcripts expressed from ΔSA containing acceptor splice site deletion are compared to those expressed from Fl and ΔL1 constructs. (C) Analysis of the intron-containing, exonized and polyadenylated transcripts in cytoplasmic and nuclear RNA fractions. Two different types of transcripts were detected with riboprobes shown above panels. Total (Σ), cytoplasmic (cyt) and nuclear (nuc) RNAs were isolated from Fl and ΔL1 transfection experiments. In experiments shown on lanes 1, 4 and 7, half of the amount of RNA was used compared to other experiments. In all experiments (A–C), transcripts detected by RPA are shown by arrows and arrowheads (faint bands) and they are schematically represented according to the scheme shown in Figure 4A. The numbers mark their sizes in nucleotides. When both, endogenous and minigene transcripts were detected, endo is shown in parenthesis. Quantitative estimation of transcripts was carried out by comparison with 2-fold serial dilution of the riboprobe shown in panel B bottom left.

The next series of results were obtained from probing transcripts, derived from the expression of transfected ABCA minigenes, with ASP and SP probes (**Figure 5A**). Properly spliced 333 nt-long L1 ASP transcript was detected for Fl, ΔSP and ΔSV40 constructs (lanes 2, 4 and 8 faint bands). An alternative 201 nt-long transcript, containing exons II and III, derived from L1 ASP using a different donor splice site [35] and showing the same protection pattern with endogenous L1 transcripts was also detected. Deletion of the L1 SP or SV40 promoter did not affect the L1 ASP transcription (cf. lanes 2, 4 and 8). It is important to note that SV40 transcription was at least 10 and 20 times more efficient than L1 SP and ASP transcription, respectively (**Figure 4C** **and** **5A**). This result is also consistent with our previous data showing that in human teratocarcinoma cells L1 SP is about twice more efficient than L1 ASP (J. Budarova and M. Speek, unpublished data). Differently from our data, in studies of Yang *et al*. [34] the L1 ASP activity was only about 1/10 of that of the L1 SP activity. This activity difference may be explained by different cell lines used (HeLa, 143B vs NTera2D1) and suggests that L1 ASP activity is higher in teratocarcinoma cells. The results of probing L1 SP transcripts were consistent with the L1 ASP transcript probing data, except that L1 SP activity seemed to depend on the SV40 transcription (**Figure 5A**, cf. lanes 11 and 17). However, the precise estimation of L1 SP activity is problematic, since the SP probe used did not differentiate between transcripts derived from L1 SP (exons SP and 3) and SV40 promoter (exons 1, 23, ExSP and 3) (**Figure 4A**). Nevertheless, from this experiment we cannot rule out that activation of L1 SP (about 2-fold) may occur by elongation of the SV40 Pol II over L1 SP region (see RT-PCR experiment above in **Figure 4B** panel 3). Similar promoter activation has been described earlier by Leupin *et al*. [18]. In addition, a small increase (about 2-fold) in L1 SP (or SV40) transcription was detected when YY1 site was deleted (cf. lanes 14 and 16). In contrast, deletion of ASP decreased SP activity about two-fold (**Figure 5A**, cf. lanes 11 and 14) suggesting that some of the TF binding sites located in ASP region were necessary for SP activity. This result is consistent with previous studies of Yang et al. [52] showing that a potential enhancer element located in L1 ASP region may increase L1 SP activity [53] (J. Budarova and M. Speek, unpublished data). Finally, it is important to note that despite the fact that the transcriptional activities of L1 SP and ASP were much lower than the activity of SV40 promoter, their effect on TI was clearly evident, as shown above (**Figure 5C**).

### L1-induced TI depends on the cryptic splice site and affects the use of polyadenylation signal in the upstream intronic sequence in ABCA minigene

Because some of the SV40 transcripts contained cryptic exon (Ex, shown in **Figure 5A**) and were prematurely polyadenylated upstream to L1 5′ UTR (determined from RT-PCR, data not shown), we decided to test whether deletion of acceptor splice site (SA) located 234 nt upstream to L1 5′ UTR could influence TI. **Figure 5B** shows that deletion of SA increases slightly the production of spliced transcripts (cf. lanes 1 and 2). However, the same deletion increases intron retention about 4-fold (cf. lanes 5 and 6), suggesting that the TI effect induced by L1 5′ UTR is strongly dependent on the presence or absence of SA in its upstream region. Analogous to the previous experiment (**Figure 4C**), deletion of L1 5′ UTR has considerably increased the transcription from SV40 promoter (cf. lanes 1 and 3, or 5 and 7).

To reveal the potential role of L1 5′ UTR in cryptic polyadenylation, the amounts of polyadenylated and non-polyadenylated transcripts were detected by polyA-containing riboprobe. **Figure 5C** shows that after deletion of L1 5′ UTR corresponding transcripts (1–23-Ex) with or without polyA disappeared, suggesting that cryptic polyadenylation as well as exonization was dependent on L1 5′ UTR (cf. lanes 1–3 and 4–6). Finally, we also determined cytoplasmic and nuclear distribution of transcripts with cryptic exon and with retained intron (**Figure 5C**, lanes 1–3 and 7–9). In all cases these aberrant transcripts were preferentially located in the cytoplasm. In summary, these results show that L1-induced TI depends on the cryptic splice site and affects the use of polyA signal in the upstream intronic sequence.

### L1-driven transcription induces exon inclusion and increases intron retention in minigene and endogeneous *NCAM1*

**Figure 6:**
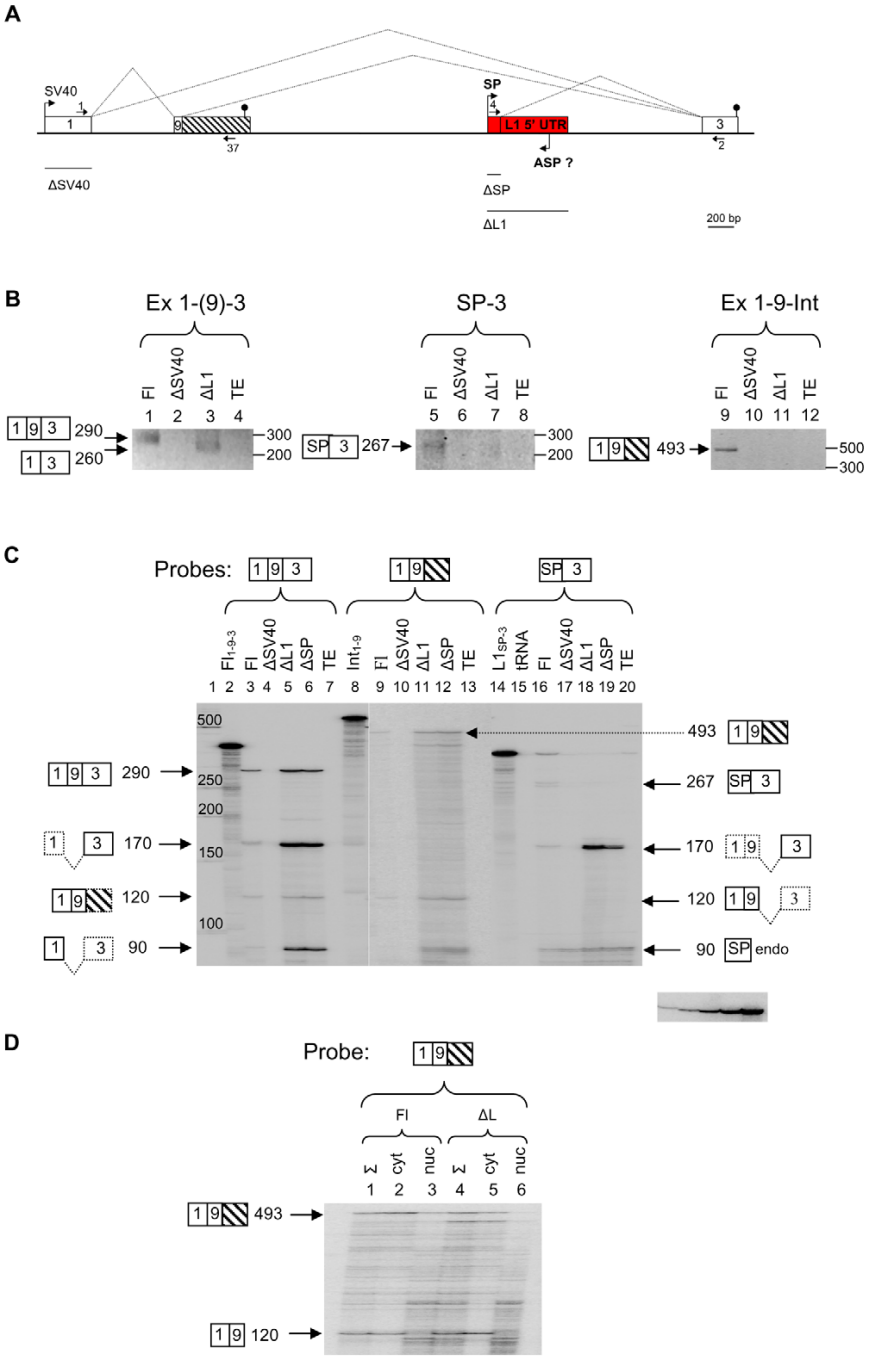
L1-induced transcription interferes with exon skipping and intron retention in NCAM minigene. (A) Schematic representation of the NCAM minigene and its deletion constructs used in transfection experiments. Deletions made in NCAM Fl construct are marked with lines below the scheme. Intron retention to exon 9 is shown by hatched box. Potential transcription from L1 ASP (although not proved by experiments, see text for details) is shown from the opposite strand. For other details see Figure 4 legend. (B) RT-PCR of various transcripts expressed from minigene constructs. Transcripts, containing exons or introns (shown on top of each panel) derived from SV40 promoter and L1 SP were detected by RT-PCR. The obtained products are shown on the left of each panel and their labeling is according to scheme A. (C) Quantitative detection of various minigene transcripts by RPA. Riboprobes Fl_1-9-3_, Int_1-9_ and L1_SP-3_ (lanes 2, 8 and 14) are schematically shown above each panel. Fl and different deletion (Δ) constructs used in transfection experiments are shown on top of each lane. Protected transcripts (marked with arrows) are schematically shown by boxes and their sizes are given in nucleotides. Dashed lines/boxes show the remaining exon(s) not protected by the riboprobe used. TE – transfection simulated with buffer. Autoradiogram clip on the bottom left shows a 2-fold serial dilution of the probe used in quantitation of transcripts. (D) Distribution of intron-containing transcripts between cytoplasm and nucleus. Total (Σ), cytoplasmic (cyt) and nuclear (nuc) RNAs were isolated from Fl and ΔL1 transfection experiments and probed with intron-containing probe.

We next used NCAM minigene to test the potential L1-induced TI predicted from the bioinformatic study. NCAM Fl was constructed by insertion of a 3636 bp genomic fragment containing intron 8, exon 9, intron 9 and L1 5′ UTR into pSPL3 vector (**Figure 6A**). This construct and various deletion constructs made from it were transfected into human teratocarcinoma cells (for preparation of constructs see Materials and Methods). After RNA isolation, transcripts were analyzed by RT-PCR and RPA. **Figure 6B** shows that deletion of L1 5′ UTR changed the splicing pattern (cf. lanes 1 and 3). While in the presence of L1 5′ UTR an Fl transcript was observed, in its absence alternatively spliced transcript without exon 9 appeared. This result suggests that L1 5′ UTR acts like a transcriptional elongation brake and facilitates exon 9 inclusion. In the next series of experiments an L1 SP-containing transcript, most likely derived from the SV40 promoter by readthrough transcription (see below), was detected only for Fl construct (lanes 5–8). Finally, intron 9 retention to spliced exons 1 to 9 was observed only in case of Fl construct, suggesting the role of L1 5′ UTR in intron retention (lanes 9–11). We have been unable to demonstrate the L1 ASP activity in NCAM minigene. Intronic probes prepared against antisense strand of NCAM intron 9 (2620 nt) yielded negative results (data not shown). However, we cannot rule out that L1 ASP-driven transcription produced spliced exons elsewhere in the NCAM structure.

Quantitative analysis of different NCAM transcripts revealed that deletion of L1 5′ UTR (or SP) increased exon 9 skipping about 5-fold (**Figure 6C**, cf. lanes 3, 5 and 6), consistent with the RT-PCR experiments (**Figure 6B**). Deletion of L1 5′ UTR resulted in a small change of the ratio between intron-containing and Fl transcripts, i.e. there were slightly more (about 2-fold) intron-containing transcripts per Fl transcripts in Fl construct experiment than in ΔL1 experiment (cf. lanes 9 and 11). Similarly to ABCA (**Figure 5A**), potential activation of the L1 SP (or exonization of this region) by readthrough transcription initiated from the SV40 promoter was observed (**Figure 6C**, cf. lanes 16 and 17). However, in this case no activity of L1 SP was detected when SV40 promoter was deleted. L1 SP also inhibited SV40 transcription, because its deletion increased SV40 promoter activity about 2-fold for Fl transcripts and about 8-fold for alternatively spliced transcripts (**Figure 6C**, cf. lanes 3 and 6). Properly spliced Fl and intron-containing transcripts were found mostly in the cytoplasmic fraction (**Figure 6D**). Our bioinformatic analysis suggested that cryptic polyadenylation in intron 9 may be due to the presence of L1. Indeed, a small increase of polyA-containing transcripts was detected for Fl transfected construct (compared to ΔL1) in RT-PCR experiment (data not shown). However, due to the very low abundance of these transcripts, we were unable to quantitate them by RPA. In conclusion, our analysis revealed that L1 5′ UTR could strongly interfere with alternative splicing and to a lesser extent affect the intron retention in NCAM.

Finally, to reveal the potential of L1-induced TI *in vivo*, we analyzed *NCAM1* transcripts derived from different cell lines and tissues (**Figure S2,** see **Figure S4**, which is a supplement to Figure S2). Our results suggest that L1 interferes with *NCAM1* transcription by causing intron retention in neuroblastoma and teratocarcinoma cell lines as well as in different human tissues.

### *KTI12*-induced transcription interferes strongly with the SV40 transcription and causes exonization and intron retention in TX-KTI minigene

**Figure 7:**
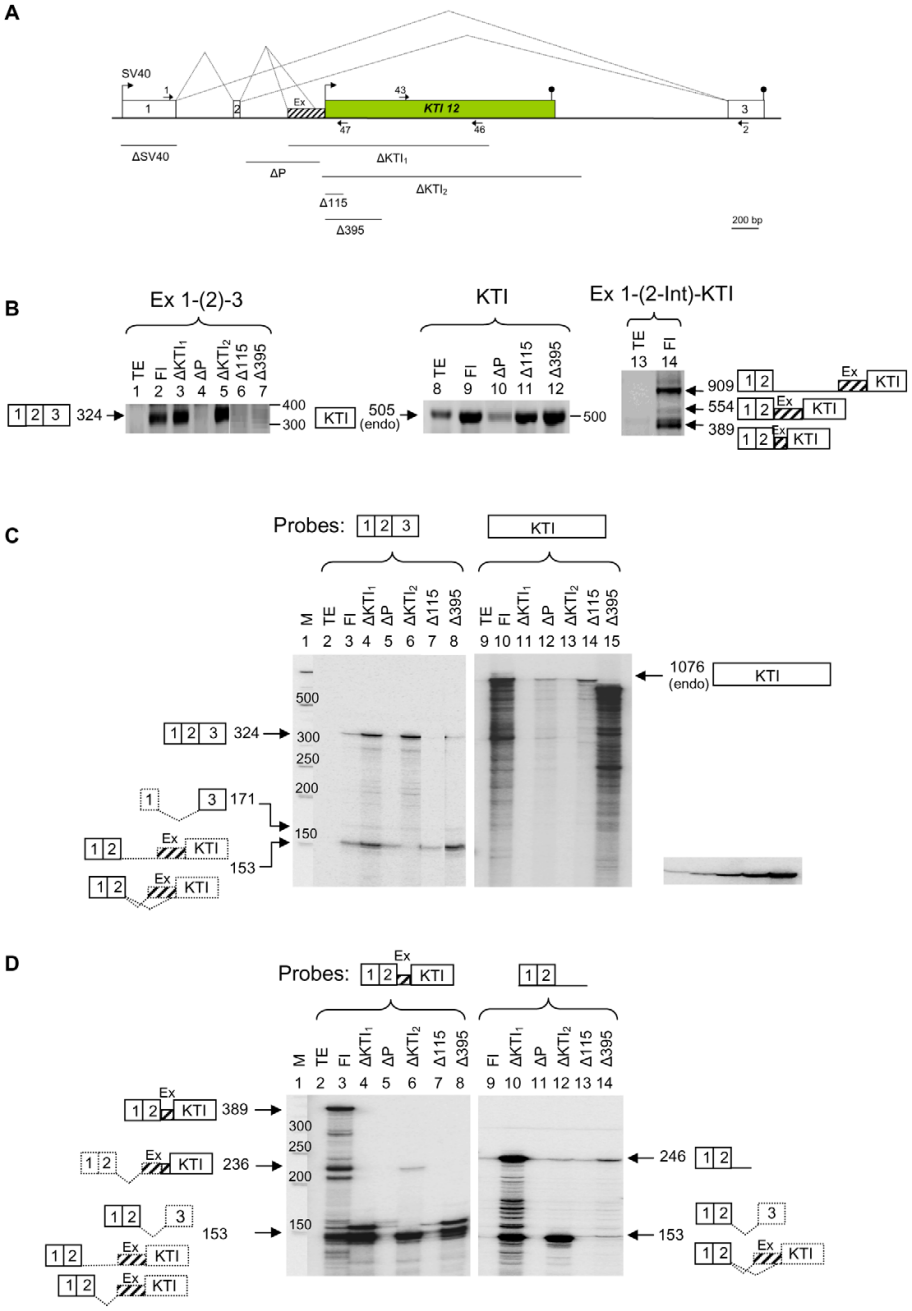
*KTI12*-induced transcription interferes with exonization and intron retention in its upstream region. (A) Schematic representation of the TX-KTI minigene and its deletion constructs used in transfection experiments. Deletions made in TX-KTI Fl construct are marked with lines below the scheme. Splicing of exons observed in different transcripts is shown by diagonal lines. Exonization of intronic sequence upstream to *KTI12* is shown by hatched box and marked with Ex. Arrows indicate the direction of transcription of SV40 and *KTI12*. (B) RT-PCR of various transcripts expressed from minigene constructs. Transcripts, derived from SV40 and *KTI12* promoters, containing exons or introns (shown on top of each panel) were detected by RT-PCR. The obtained products are shown on the left of each panel and their labeling is according to the scheme A. (C) Quantitative detection of various minigene transcripts by RPA. Riboprobes are schematically shown above each panel. Fl and different deletion (Δ) constructs used in transfection experiments are shown on top of each lane. Protected transcripts (marked with arrows) are schematically shown by boxes and their sizes are given in nucleotides. Dashed lines/boxes show the remaining exon(s) not protected by the riboprobe used. The smear of partially protected fragments (shown on lanes 10 and 15) most likely corresponds to prematurely terminated transcripts. TE - transfection simulated with buffer. Autoradiogram clip on the bottom right shows a 2-fold serial dilution of the probe used in quantitation of transcripts. (D) Detection of various TI transcripts. Intron-containing riboprobes used are shown above each panel. Protected transcripts are schematically shown as in (C).

Using the third minigene, we investigated how a single exon nested gene *KTI12* can affect transcription of its host gene *TXNDC12*. TX-KTI Fl minigene construct was made by insertion of a 226 bp genomic fragment of *TXNDC12* containing exon 2 and a 2367 bp *KTI12* coding region together with their 5′ and 3′ flanking sequences into exon trapping vector pSPL3 (**Figure 7A**). In this construct, SV40 promoter was arranged tandemly with respect to the *KTI12* transcription. To investigate the effect of different transcription units to TI, several deletion constructs were prepared (**Figure 7A** **,** for details see Materials and Methods). All constructs were transfected into human teratocarcinoma cells and their expression was analyzed by RT-PCR and RPA. In Fl, ΔKTI_1_ and ΔKTI_2_ transfection experiments, the presence of a 324 nt SV40 transcript containing exons 1, 2 and 3 was detected (**Figure 7B**, lanes 2, 3 and 5). In this case, a small increase in SV40 transcription observed for deletion constructs suggests that *KTI12* decreases its transcription. Surprisingly, however, deletions made in the upstream and initiaton regions of *KTI12* yielded almost no SV40 transcripts (lanes 4, 6 and 7), indicating that these regions are required for efficient SV40 transcription.

In the following series of RT-PCR experiments transcripts derived from *KTI12* were determined. These transcripts and less abundant endogenous transcripts of the expected size (505 nt), encompassing *KTI12* positions 595–1099, were detected for all four constructs (**Figure 7B**, lanes 8–12). Deletions made in the initiation region (Δ115 and Δ395) did not affect the overall transcription of *KTI12* (cf. lanes 9, 11 and 12). However, deletion in a putative promoter region of *KTI12* showed transcription level comparable to that of TE, suggesting endogenous transcription (cf. lanes 8 and 10). In addition, *KTI12* transcription was detected for ΔSV40, indicating that *KTI12* activity was independent from SV40 promoter activity (data not shown). Consistent with our bioinformatic study, a 554 nt-long SV40 transcript containing exons 1, 2 and a cryptic exon (Ex) generated by using an acceptor splice site 251 nt upstream to the most 5′ transcription start site of *KTI12* was detected (lane 14). Two additional transcripts, a 389 nt transcript contained alternative acceptor splice site (86 nt upstream to *KTI12*) and a 909 nt transcript, derived from the readthrough of the intron between exon 2 and *KTI12* were also detected. Therefore, these results suggest that *KTI12* decreases the efficiency of SV40 transcription by forcing exonization using alternative cryptic acceptor splice sites located upstream to *KTI12*.

To further investigate the TI effects observed in RT-PCR experiments, we next used RPA. The following results were obtained from probing transcripts expressed from transfected minigenes using Fl and KTI riboprobes. Deletion of *KTI12* (coding region in ΔKTI_2_, including promoter region in ΔKTI_1_) increased SV40 transcription about 5-fold, suggesting that *KTI12* interferes strongly with the elongating Pol II (**Figure 7C**, cf. lanes 3, 4 and 6). The ratio between transcripts containing exonized region (1–2-Ex) and those contaning exons 1–2–3 (Fl) was higher (about 2-fold) in Fl minigene than in ΔKTI_1_ (cf. lanes 3 and 4) suggesting that *KTI12* interferes with intron retention and forces exonization in its upstream region. Deletion of *KTI12* (ΔKTI_2_, lane 6) eliminated these effects, thus further supporting this conclusion. Consistent with RT-PCR results described above (**Figure 7B**), ΔP, Δ115 and Δ395 showed minimal SV40 transcription across exons 1, 2 and 3 (**Figure 7C**, lanes 5, 7 and 8). However, the same deletions showed transcription and splicing of exons 1 and 2 comparable to that of the Fl construct (cf. lanes 3, 5, 7 and 8). Also, for these deletions, variable level of readthrough or initiation of transcription from *KTI12* was observed (lanes 12, 14 and 15) ranging from low level or endogenous transcription for ΔP to high level for Δ395. Deletions made in the initiation region (Δ115 and Δ395) did not abolish the *KTI12* transcription (lanes 14 and 15) suggesting the redundancy in transcriptional initiation. Heterogeneous transcription initiation from about 300 nt interval was also supported by ESTs (data not shown).

It was expected that deletion of *KTI12* promoter region (ΔP) would increase SV40 transcription. However, no such increase was observed. Instead, about 2-fold decrease in transcription was detected, while the amount of intron-retained and exonized transcripts remained unchanged (**Figure 7C**, cf. lanes 3 and 5). This result shows that *KTI12* promoter region somehow contributes to the SV40 transcription, however, the reason for this remains unclear.

To further analyze the essence of non-Fl transcripts shown in **Figure 7C**, two riboprobes complementary to transcripts containing cryptic exons and intron were used (**Figure 7D**). Surprisingly, forced exonization appeared almost exclusively in Fl construct (cf. lanes 3 and 6–8). In this case both acceptor splice sites were almost equally used. A minimal level of the exonization was also detected for ΔKTI_2_ construct (lane 6). The ratio of Fl transcripts to intron-containing transcripts was comparable in Fl, Δ115 and Δ395 (lanes 9, 13 and 14), but it was significantly increased in ΔKTI_2_, (cf. lanes 9 and 12) suggesting that *KTI12* contributed to the intron retention. The high level of intron retention in ΔKTI_1_ (lane 10) most likely reflected transcriptional readthrough.

In summary, despite the fact that in some deletion experiments the contribution of *KTI12* promoter and initiation region to TI was difficult to interpret, our data support the conclusion that *KTI12* interferes strongly with SV40 transcription by forcing exonization and causing intron retention in its upstream region. All these data suggest that, similarly to L1, a tandemly located nested gene interferes with the transcription and splicing of the host gene.

## Discussion

**Figure 8:**
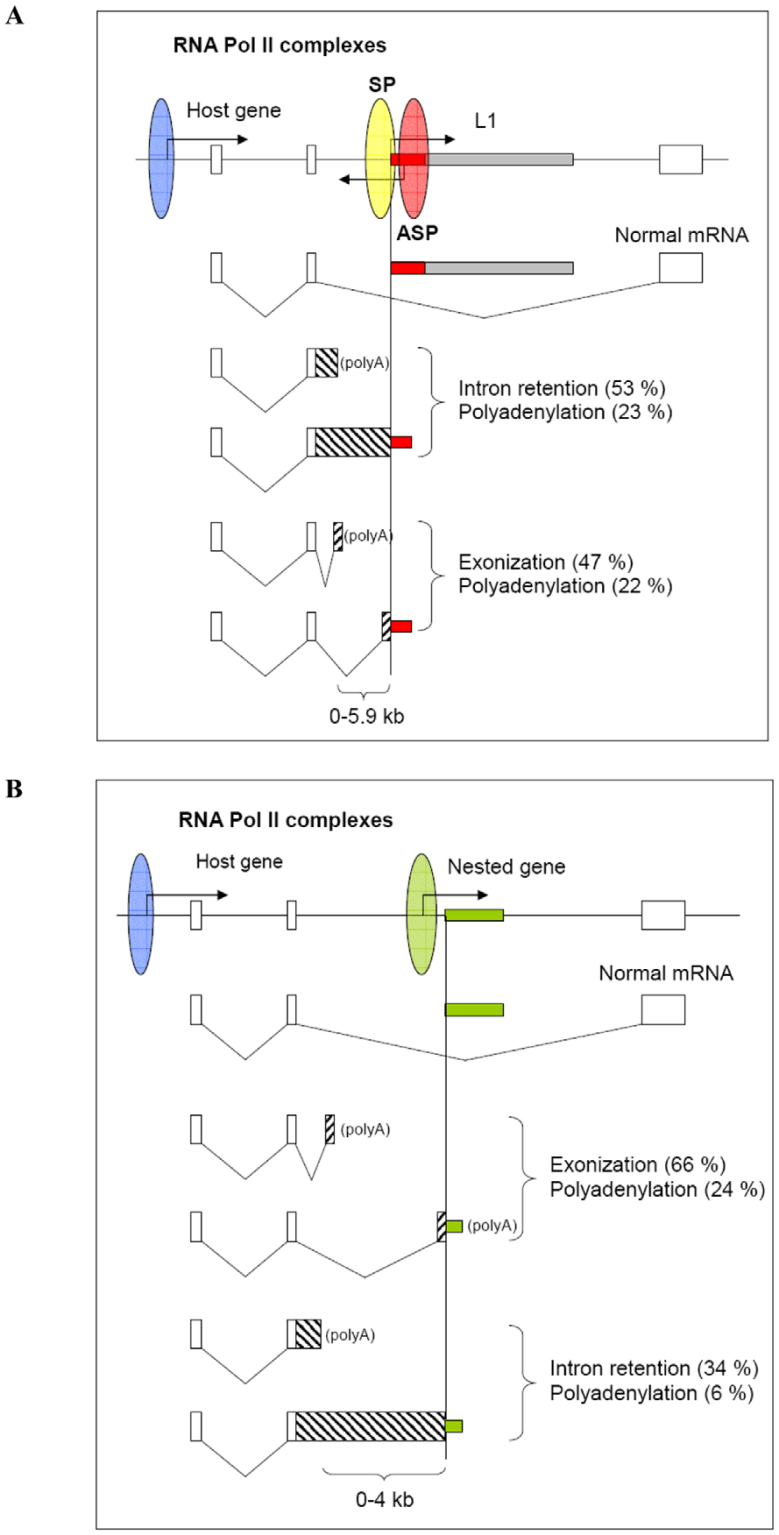
TI effects induced by human L1 retrotransposon and nested gene. (A) Tandemly arranged intronic L1 interferes with the host gene transcriptional elongation by forcing intron retention, exonization and cryptic polyadenylation. The TI effect of L1 depends on its SP and ASP activity, cryptic splice sites and polyA signals located upstream to L1. (B) A tandemly arranged nested gene interferes with the host gene transcription by causing exonization and intron retention in its upstream region. Pol II complexes are shown with ellipses (host in blue, nested in green and L1 SP and ASP in yellow and red, respectively) and their direction of transcription with arrows. Exons are displayed with boxes and introns with lines. Splicing is shown by diagonal lines. Intron retention and exonization are shown with hatched box with downward and upward diagonals, respectively. Frequency (%) of exonization, intron retention and polyadenylation induced by L1 5′ UTR and protein-coding and nc nested genes was determined from the data shown in Table S1 and Table S3.

In this paper, we show that TI induced by L1s and nested genes may be characterized by three different effects: intron retention, forced exonization and cryptic polyadenylation (**Figure 8**). It is important to note that these effects, first experimentally determined from the endogenous transcripts and and then proved from the expression of three minigenes, were accurately predicted from our initial bioinformatic approach. TI induced by L1 was most efficient in ABCA minigene, showing about 8-fold increase in SV40 transcription and splicing upon deletion of L1 5′ UTR. On the contrary, the presence of L1 5′ UTR inhibited SV40 transcription and forced intron retention (**Figure 4**). Both, L1 SP and ASP contributed to the TI. Although the effect of L1 SP was greater than that of ASP, a small synergy between them was observed. Since the net effect of L1 SP and ASP deletions was not equal to the deletion of the entire L1 5′ UTR, it seems likely that other regions in the L1 5′ UTR (positions +96 to +306 and +599 to +990), containing binding sites of unknown TFs [30], [52], [54], also contributed. Consistent with previous studies of others [37], [38], [48], [51], [55], full-length L1 RP (or L1 PA3) and to a lesser extent L1 ORFs in sense orientation reduced significantly SV40 transcription, suggesting that both L1 5′ UTR and ORFs contributed to TI. Also, in some studies [38], [51], [55] 5′ truncated L1s (more frequent forms) proved to be much less effective in inhibition of transcription suggesting that maximal TI effect will be obtained with full-length L1.

In ABCA minigene, the presence of cryptic acceptor splice site upstream to L1 5′ UTR determined the efficiency of L1-induced TI, because its deletion increased intron retention about 5-fold. Therefore, the occurrence of cryptic acceptor splice sites is somehow beneficial for SV40 Pol II to guarantee processive transcription coupled with splicing across exons 23, ExSP and 3 (**Figure 4A**), whereas the absence of it may force Pol II to slow down or even dissociate from the template, giving rise to premature intron-containing transcripts (**Figure 8A**). Alternatively, elongating Pol II could search for additional, less favourable splice sites, causing exonization in upstream region. All these data are consistent with the kinetic model of transcription originally proposed by Eperon *et al*. [56] and modified by Kornblihtt [57]. According to this model, the use of alternative splice sites depends on the elongation rate of Pol II. In our minigenes, L1 SP Pol II complex could act as a roadblock (or sitting duck) by forcing elongating SV40 Pol II to pause or dissociate from the template. Since L1 ASP Pol II complex is moving in opposite direction, it could collide with SV40 Pol II complex and decrease its efficiency of transcription. The fact that L1 SP and ASP activities are significantly lower than SV40 promoter activity does not necessarily mean that their contribution to TI is minimal. It is possible that the binding affinities of L1-specific TFs and cooperativity between them will determine the net effect on TI. The TI effect may also depend on the promoter strength, which, according to a recent study [58] can be determined by the balance between productive and abortive initiation of transcription. It is reasonable to assume that both, productive as well as abortive transcription (resulting <15 nt transcripts), could contribute to TI. Moreover, binding of TFs alone without initiation of transcription could affect the outcome of TI. It may be argued that an interplay between L1 SP and ASP could influence the SV40 promoter-driven transcription and splicing in some complicated or combinatorial way, which is not so easy to trace from simple deletion analysis. However, it is clear that deletion of the L1 5′ UTR or part of it in ABCA minigene affects SV40 transcription strongly. Nevertheless, we cannot completely rule out the possibility that other factors (sequence composition or nucleotide bias) could also contribute to the net TI effect, similar to that described earlier [59], [60].

Consistent with pausing of transcriptional elongation induced by L1 5′ UTR, we observed cryptic polyadenylation in ABCA. This effect was solely dependent on the presence of L1 5′ UTR (**Figure 5C**). Therefore, not only the presence of cryptic acceptor splice site, but also the presence of cryptic polyA signal upstream to L1 can have an impact on the premature transcriptional termination. Previously, highly similar effect induced by IAP retrotransposon in *Cabp* was observed [17]. In addition, cryptic polyadenylation within the L1 ORF1 and ORF2 sequences was observed by two groups [37], [38]. The important novel aspect here is that L1 5′ UTR could also interfere with polyadenylation in its upstream region. In addition, the L1-induced TI could be distance dependent (see below).

Differently from ABCA, the main TI effect observed in NCAM minigene studies was the inclusion of exon 9. Deletion of the L1 5′ UTR facilitated transcriptional elongation across intron 9, resulting in exon skipping consistent with the kinetic coupling model [57]. Clearly, the difference between ABCA and NCAM minigenes may be explained by the absence of proper acceptor splice site upstream to L1 5′ UTR and the longer distance between upstream exon and L1 5′ UTR in NCAM (2620 vs 559 nt). Similar to our NCAM minigene studies, the influence of intronic Alu elements on the mode of alternative splicing (constitutive vs alternative) has been studied [61], [62]. It was found that Alus located in intron in antisense orientation are capable for influencing alternative splicing of flanking exons by two different mechanisms. In contrast, our preliminary studies showed that, similar to L1s, Alus in sense orientation located close to exons could also influence their exonization (data not shown).

How many L1s could possibly cause TI in the human genome? Of the total of 7000 full-length L1s [26], half of which are located in introns of genes [27], roughly 1000 have tandem arrangement with the host genes [63]. Using next generation sequencing, Rangwala *et al*. [29] have determined transcriptional activity of 232 full-length L1s in human lymphoblastoid cell lines. Of these, younger L1 families (PA1–PA5) were overrepresented, compared to older families (PA6-PA7). This result is in accordance with the conservation of TF binding sites in L1 PA1-PA7 [26]. The latter authors also showed the conservation of L1 ASP in L1 PA1-PA6 families. This is consistent with our earlier data [36], conservation of transcriptional activity of L1 ASP [64] and our genome-wide analysis, showing that 50 L1s (PA1–PA7, plus one PA8 and PA10) are possibly involved in TI (**Table S1**). However, this number is clearly an underestimate and does not consider inter-individual variation and expression differences in different cell types or tissues (discussed in [29]). In this context it is important to note that Faulkner *et al*. [28] have recently mapped thousands of gene expression tags to L1 SP and ASP regions and their data suggest that a large number of L1s (possibly thousands) could contribute to the human transcriptome. It is also possible that many aberrant transcripts generated by TI may remain undetected due to mRNA surveillance or nonsense-mediated decay [65], although the two minigene transcripts with retained introns analyzed here were found mostly in the cytoplasmic fraction (**Figure 5C** **and** **6D**). In addition, in our search, we considered spliced transcripts containing introns or exonized introns as potential TI candidates. However, we also noticed that at least the same number of L1s had intronic fragments in their immediately upstream region, and frequently contained DNase hypersensitive sites and H3K27Ac histone marks [66], suggesting transcriptional activity in this region. Therefore, we predict that a minimum of 100 full-length intronic L1s (most likely L1 PA1–PA7) may be involved in the TI of host genes.

Differently from the intronic L1s, which showed comparable intron retention and exonization effects, the main effect determined for nested protein-coding (including *KTI12*) and nc genes was exonization (**Figure 8B**). Interestingly, cryptic polyadenylation upstream to the nested genes was observed mostly in transcripts undergoing forced exonization. It is likely that at least some nested genes have coevolved to an extent to maintain coexpression. Still, it is unclear, whether exonization of intronic sequence upstream to a single exon nested gene is required for their normal expression or whether it is a part of misregulation between these genes. Answer to this question might come from the analysis of the TI effects in normal and tumor tissues. Of the 104 nested genes analyzed, 66 (63 %) and 22 (21 %) showed TI effect in normal and tumor (transformed cell lines) tissues, respectively (**Table S3**). And 16 (16 %) nested genes showed TI ESTs in both types of tissues. Similar preferential expression in normal tissues was observed for L1 (**Table S1**). We speculate that about 3-fold preferential expression in normal tissues indicates that TI between nested genes may be part of their normal regulation, although the possibility of misregulation in some cases cannot be excluded. Our results also suggest that TI between nested genes may be widespread and frequently restricted to different human tissues, although we cannot exclude that for some genes tissue-specific patterns (depending on the TF binding or promoter activity) may be observed.

In this study, several lines of evidence suggest that L1 or nested gene is able to cause TI in normal gene transcription. Not only ESTs, showing unequal distribution in introns of host genes, but also the orientation bias (tandem orientation being twice less frequent than convergent orientation) [23], [51], [63] suggests the negative impact of L1. We also noticed that the distance between upstream exon of host gene and L1 (0–5.9 kb) or the nested gene (0–4.0 kb) may be an important determinant of the TI effect. For instance, 40 % of the intronic L1s were causing TI effect within 0.5 kb distance upstream to L1, compared to 52 % of protein-coding and nc nested genes (**Figure S3**). Interestingly, Zhang *et al.* [67] have recently shown that the proportion of transposable elements (Alu, L1, LTR-containing retroelements) contributing to aberrant or chimeric transcripts is significantly higher within so called hazardous zones (<5 kb). Therefore, it may be that distance-dependent sterical hindrance between Pol II complexes is dictating the efficiency of TI. Whether or not this is the case, a detailed analysis (the presence or absence of cryptic splice sites and polyA signals) is required to establish the putative TI effect in each individual case.

Although, in our experiments, we were unable to distinguish between the DNA binding of TFs and the act of transcription as a cause of TI effect in nested genes, it seems likely that DNA-bound TFs may play a role in reducing the speed of transcription elongation. In support of this kinetic model [57], TF binding to many nested genes and repeated DNAs was detected in chromatin immunoprecipitation experiments [66]. Nevetheless, further analysis is required to differentiate between DNA binding and transcription.

L1 retrotransposons have been shown to influence the expression of host genes by variety of effects, including insertional mutagenesis, mobilization and transcriptional regulation of cellular genes [68]. We believe that the TI determined in this study adds another layer of complexity and will further expand our understanding in the transcriptional regulation between host genes and L1 retrotransposons as well as between nested genes.

### Conclusions

We have characterized TI between host genes and intronic L1 retrotransposons as well as between the nested genes. Based on our bioinformatic study, we first predicted the existence of TI between tandemly arranged host genes and intronic L1 retrotransposons and nested genes, and thereafter proved it experimentally using the expression of three different minigenes in cell culture. Analysis of endogenous transcripts derived from different human cell lines and tissues also supported TI between protein-coding and nc nested genes. Our results suggest that TI induced by intronic L1s and nested genes may be widespread and could influence the expression of many genes. Further analysis of TI effects between nested genes should reveal their role in the expression of a large number of host genes in normal as well as in tumor tissues. Also, it would be interesting to determine whether other retroelements (endogeneous retroviruses, Alus, SVAs, etc) could exert similar TI effects as L1 retrotransposons and whether their effects may be due to DNA-bound TFs not just transcriptional activity or the mere presence of splicing and polyadenylation signals.

## Materials and Methods

### Bioinformatics

*In silico* chromosome walking with 100 kb steps was carried out on the human genome with UCSC Genome Browser [39] using March 2006 (hg18) assembly (NCBI Build 36.1). Upstream regions of nested genes or intronic L1s were analyzed for aberrant ESTs/mRNAs of host genes showing intron retention and/or exonization to spliced exons and cryptic polyadenylation. Exon-intron structure of the host and nested genes or intronic L1s was analyzed by Spidey [69]. Splice sites were predicted with NNSPLICE 0.9 version [70] and polyA signals with POLYADQ [71]. DNA repeats were determined by Repeatmasker (A.F.A. Smit, R. Hubley & P. Green, unpublished data). Tissue-specific expression of the host and nested genes was determined by using NCBI UniGene's EST Profile Viewer.

### Genomic PCR

A 1036 bp *ABCA9* fragment containing exon 23 (108 bp) and its 5′ and 3′ flanking regions (402 and 526 bp, respectively) was amplified from genomic DNA derived from NTera2D1 cell line using PCR with primers 22 int Dir and 23 int Rev. Similarly, *NCAM1* genomic region was amplified in three overlapping fragments. First, a 1733 bp fragment A containing exon 9 (30 bp) and its flanking regions was amplified with primers 8 int Dir and 9 int Rev 2. Second, a 1908 bp fragment B containing intron 9 was amplified with primers 9 int Dir 2 and 9 int Rev 1. Third, a 848 bp fragment C containing intron 9 and L1 5′ UTR was amplified with primers L1 Dir and L1 Rev. Fragments A, B and C were used to restore a 3637 bp *NCAM1* genomic region containing L1 5′ UTR (see Minigene constructs). *TXNDC12-KTI12* genomic region was amplified in two fragments. A 226 bp *TXNDC12* fragment containing exon 2 (61 bp) and its 5′ and 3′ flanking intronic sequences (71 bp and 93 bp, respectively) was amplified with primers TX ex Dir and TX ex Rev. A 1896 bp *KTI12* and its 5′ and 3′ flanking sequences (478 bp and 193 bp, respectively) was amplified with primers KTI Dir 2 and KTI Rev. PCR was carried out with recombinant *Taq* DNA polymerase (Fermentas) according to manufacturer's protocol. For cloning, genomic fragments were treated with Klenow polymerase (Fermentas) and purified by TAE agarose gel electrophoresis using InvisorbSpin kit (Invitek). All primers used in this study were purchased from TAG Copenhagen A/S and their sequences are listed in **Table S5**.

### Minigene constructs

*ABCA9, NCAM1* (fragments A, B and C) and *TXNDC12* gel-purified genomic fragments were first cloned into pBlueScript (pBS) SK^+^ vector (Stratagene) digested with *Eco*RV. *KTI12* genomic fragment was cloned into pBS KS^+^ vector (Stratagene) digested with *Sma*I. Positive recombinant plasmids were isolated with alkaline lysis minipreparation method [72].

L1 5′ UTR-containing #11AS fragment (990 bp) was obtained from a recombinant pGL3 vector [31] digested with *Ecl*136II and *Hind*III, treated with Klenow polymerase and cloned downstream to the *ABCA9* insert in pBS SK^+^ vector blunted at *Cla*I site. From this plasmid, *ABCA9*-L1 5′ UTR fragment was separated after digestion with *Sal*I, then treated with Klenow polymerase and digested with *Eco*RI. The obtained fragment was further cloned into exon trapping vector pSPL3 (Invitrogen), from which a 1 kb intron fragment containing cryptic splice site at position 1134 bp was removed with *Nhe*I, after blunting the ends with Klenow polymerase and digestion with *Eco*RI, thus leaving appropriate sites (blunt and *Eco*RI) for its insertion. The final plasmid was termed ABCA Fl (7.2 kb) and contained SV40 promoter-*ABCA9*-L1 5′ UTR-SV40 polyA signal (**Figure 4A**). The entire L1 5′ UTR deletion (990 bp) was made in the ABCA Fl with *Xho*I and *Nhe*I generating ΔL1. A 292 bp and 613 bp deletions in L1 ASP (L1 5′ UTR positions +307 to +599 and +384 to +993, respectively), named ΔASP_292_ and ΔASP_613_, were made with *Bsp*T1 and *Nhe*I, respectively. ΔYY1 (deletion +13 to +21 in L1 5′ UTR) was generated by cloning a 132 bp fragment, starting with YY1 primer sequence and ending with *Pau*I site (obtained from PCR with primers YY1 and L1 ASP10) into ΔASP_292_ or ABCA Fl construct between blunted *Xho*I and *Pau*I site. A 96 bp deletion (positions +1 to +96) in L1 SP was made with *Xho*I and *Kpn*I and the construct was named ΔSP. Double deletions were made consecutively. A 2 bp deletion (ag) in the acceptor splice site (ΔSA) in intron 23 in ctttccagATGGTGCTAAA was made in the ABCA Fl with PCR mutagenesis. First, PCR was carried out with two primer pairs: 23 ex Dir – SA mut2, and SA mut1 – L1 SF1. The obtained two fragments were combined and amplified with primers 23 ex Dir and L1 SF1. Then, the PCR product was digested with *Acc*65I and inserted into ABCA Fl from which the same, but nonmutated fragment (740 bp) was removed. Sox mutations in the ABCA Fl (positions +472 to +477 and +572 to +577 in respect of L1 5′ UTR sequence [33]) were also made with PCR mutagenesis as follows. PCR was carried out with four primer pairs: BspTI Dir – Sox1 Rev / Sox1 Dir – BspTI Rev for a Sox1 and BspTI Dir – Sox2 Rev / Sox2 Dir – BspTI Rev for a Sox2 mutation. The obtained two fragments for both mutations were combined and amplified with primers BspTI Dir – BspTI Rev. For Sox1/2 mutations two fragments were amplified from combined Sox1 template with primer pairs BspTI Dir – Sox2 Rev and Sox2 Dir – BspTI Rev, fused and amplified with BspTI Dir – BspTI Rev. Then the mutated PCR products (Sox1, Sox2 and Sox1/2) were digested with *Bsp*T1 and inserted into ABCA Fl from which the same, but nonmutated fragment (292 bp) was removed. A 423 bp deletion in SV40 promoter (positions 68–491 nt) in ABCA Fl was made with *Pae*I and *Nco*I generating ΔSV40. Similarly, partial SV40 promoter deletion (positions 68–140 nt) was made with *Pae*I generating ΔSV40_part_.

A 960 bp L1 PA3 5′ UTR fragment from *ABCA9* locus was amplified from genomic DNA derived from HeLa cell line using PCR with primers L1 PA3 5′ UTR Dir and L1 5′ UTR Rev. RP 5′ UTR (903 bp), RP ORFs (5121 bp) and RP 5′ UTR+ORFs fragments (6006 bp) were amplified from L1 RP clone (AF148856, kindly provided by Richard Badge) with L1 RP 5′ UTR Dir – L1 5′ UTR Rev, L1 RP ORF1 Dir – L1 3′ UTR Rev and L1 RP 5′ UTR Dir – L1 3′ UTR Rev primers, respectively. We were unsuccessful in amplification of the full-length L1 PA3 from the *ABCA9* locus, because of high content of repeated DNAs in the 3′ flanking region. Therefore, we fused L1 5′ UTR with RP ORFs which were 98% identical to L1 PA3 ORFs. Obtained fragments were treated with Klenow polymerase and cloned into pBS SK^+^ vector blunted at *Eco*RV site with different orientation. Thereafter these fragments were digested from pBS SK^+^ with *Sal*I and *Sma*I and subcloned downstream to the *ABCA9* insert into ABCA Fl, from which #11AS 5′ UTR was removed with *Nhe*I, after blunting the ends wiht Klenow polymerase and digestion with *Xho*I. These constructs were named PA3 5′ UTR, RP 5′ UTR, RP ORFs +, RP ORFs –, RP 5′ UTR+ORFs +, RP 5′ UTR+ORFs – and PA3 5′ UTR+ORFs.

*NCAM1* genomic fragments A, B and C were ligated together as follows. Fragments A and B obtained from the appropriate recombinant pBS SK^+^ digested with *Bgl*II and *Sma*I were ligated and inserted into pBS SK^+^ digested with *EcoR*V. Fragment C, obtained after digestion with *Hind*III, blunted with Klenow polymerase and digested with *Spe*I, was inserted downstream to the fragments A–B using *Spe*I and *Not*I, blunted with Klenow polymerase. Finally, a 3637 bp *NCAM1* genomic fragment cloned in pBS SK^+^ was digested with *Sac*II, blunted with Klenow polymerase and digested with *Sal*I. This fragment was further cloned into exon trapping vector pSPL3 from which a 1 kb intron fragment was removed with *Nhe*I as described above. The final plasmid was termed NCAM Fl (8.7 kb) and contained SV40 promoter-*NCAM1*-L1 5′ UTR-SV40 polyA signal (**Figure 6A**). A 156 bp deletion was made in NCAM Fl with *Spe*I and *Acc*65I. This deletion removed L1 SP (L1 5′ UTR positions +1 to +97) generating ΔSP. ΔL1 was made by removing a 3249 bp fragment (A–B) from NCAM Fl construct with *Spe*I and *Sal*I. After blunting the ends, this fragment was inserted into pSPL3 vector digested with *Nhe*I and *Xho*I. A 611 bp deletion of SV40 promoter (positions 74–685 nt in pSPL3) was made in NCAM Fl with *Sal*I and *Mph*1103I generating ΔSV40.

A 273 bp *TXNDC12* genomic fragment, containing exon 2 obtained from recombinant pBS SK^+^ after digestion with *Xho*I and *Sma*I, was cloned upstream to *KTI12* in pBS KS^+^ digested with *Xho*I and *Eco*RV site in the same transcriptional orientation. From this cloning a 2674 bp *TXNDC12-KTI12* fragment obtained after digestion with *Xho*I and *Xba*I was inserted into exon trapping vector pSPL3 digested with the same restriction enzymes. As described above, from this vector a 1 kb intronic fragment was removed before cloning. The final plasmid was termed TX-KTI Fl (7.7 kb) and contained: SV40 promoter-*TXNDC12*-*KTI12*-SV40 polyA signal (**Figure 7A**). A 1677 bp deletion was made in TX-KTI Fl with *Pst*I. This deletion removed putative promoter and about 2/3 of the initiation and coding region of *KTI12* generating ΔKTI_1_. A 492 bp deletion was made in the upstream region of *KTI12* with *Mlu*I and *Eco*RI (partial digestion) generating ΔP. The entire *KTI12* coding sequence (1910 bp) was removed with *Mlu*I and *Bam*HI generating ΔKTI_2_. A 115 bp (Δ115) and 395 bp (Δ395) deletions were made within the initiation region of *KTI12* with PCR deletion mutagenesis, similarly as described above (ABCA ΔSA) using TX-KTI pBS construct and two primer pairs: T3 – KTI Δ1 as2 and KTI Δ1 s2 – KTI Rev RT in the case of Δ115 and T3 – KTI Δ2 as and KTI Δ2 s – KTI Rev RT in the case of Δ395. PCR fragments were purified from agarose gel and amplified with fusion PCR with T3 and KTI Rev RT primers. After digestion with *Xho*I and *Kpn*I these fragments were gel-purified and cloned into pSPL3 vector digested with the same restriction enzymes. Deletion of SV40 promoter (positions 72–685 in pSPL3) in TX-KTI Fl was made with *Pae*I and *Sal*I generating ΔSV40.

In cloning experiments, where deletions were made with heterologous restriction enzymes, the ends were blunted with Klenow polymerase and ligated using T4 DNA ligase (Fermentas). Restriction fragments were separated and purified from TAE gel with InvisorbSpin (Invitek) kit. Structures of the final plasmid DNAs were confirmed by restriction mapping and sequencing using BigDye Terminator Cycle Sequencing Kit (Applied Biosystems) according to the manufacturer's protocol. Sequences of all Fl minigene constructs are available upon request.

### Cell culture and DNA transfection

Human teratocarcinoma (NTera2D1, ATCC number: CRL-1973), adenocarcinoma (HeLa, ATCC number: CCL-2), and mouse neuroblastoma (N2A, ATCC number: CCL-131) were purchased from LGC Standards. Human neuroblastoma (Kelly) was a gift from A. Veske. Cells were grown at 37°C in an atmosphere containing 5% carbon dioxide (CO_2_) in Dulbecco's modified Eagle medium (DMEM) supplemented with 10% fetal bovine calf serum, penicillin (100 u/ml) and streptomycin (100 µg/ml) (Invitrogen). Cells were plated in 6-well tissue culture dishes with appropriate density 24 hour before transfection. Transient transfection was carried out with calcium phosphate [73] using 2.5 µg of plasmid DNA per well. TE (10 mM Tris-HCl, pH 7.5, 1 mM EDTA-Na_2_) was used as a negative control. All transfections were made in three parallels. After 4 hours, transfection mix was replaced by fresh medium and incubation continued 24–40 hours.

### RNA isolation

Total RNA was isolated from transfected or nontransfected cells using TRIzol reagent (Invitrogen), extraction with chlorophorm and precipitation with isopropanol as described by the manufacturer's protocol. The RNA pellet was washed with 70% ethanol, dissolved in PK buffer (100 mM Tris-HCl, ph 7.5, 0.22 M NaCl, 1% SDS and 12.5 mM EDTA-Na_2_) and incubated with proteinase K (Fermentas) (0.2 µg/µl) at 37°C for 2 hours. After incubation, RNA was treated with phenol and precipitated with ethanol. DNA was removed from RNA samples by DNase treatment (0.05 u/µl DNase I, Fermentas) at 37°C for 2 hours. Finally, RNA was treated with phenol, precipitated with ethanol, dissolved and kept in TE at −20°C. The amount and quality of RNA was checked by electrophoresis of one tenth of RNA sample.

Cytoplasmic and nuclear RNA fractions were isolated from Kelly and NTera2D1 cell lines using the following protocol. Cells were washed with PBS, placed on ice and lysed directly on dish with 1 ml of NP-40 lysis buffer containing 0.5% Nonidet P-40, 3 mM MgCl_2_, 10 mM NaCl, 10 mM Tris-HCl, ph 7.4 and 1 mM DTT. After incubation for 5 min, lysed cells were removed and transfered to centrifuge tubes. Nuclei were pelleted at 4°C by centrifugation at 3000 rpm for 3 min. Supernatant containing cytoplasmic RNA was extracted with phenol and precipitated with ethanol. Nuclei were suspended in 300 µl ice-cold NP-40 lysis buffer, pelleted by centrifugation and suspended in 50 µl lysis buffer. Nuclear RNA was isolated with TRIzol reagent as described above. All RNA fractions were treated with DNase and proteinase K, dissolved in FA hybridization cocktail (40 mM PIPES, pH 6.4, 1 mM EDTA, 0.4 M NaCl and 80% formamide) and kept at −20°C.

### RT-PCR

Reverse transcription (RT) was performed essentially as follows, 0.2–1.0 µg of total RNA was hybridized with appropriate primers: pSPL3 Rev primer for SV40 and L1 SP transcripts; 22 int Dir primer for ABCA L1 ASP transcripts; 23 int Rev primer for ABCA SV40 intronic transcripts; oligo dT_AGC_ primer for SV40 exonization and polyadenylated transcripts in ABCA Fl and NCAM Fl; 9 int Rev B primer for NCAM 1–9 transcripts; KTI Rev RT primer for *KTI12* transcript; EST1 Rev for SV40 exonization transcripts in TX-KTI Fl, incubated at 65°C for 5 min and chilled on ice. RT reactions were carried out with SuperScript III RTase (Invitrogen) (final concentration 5 u/µl) in a final volume of 5 µl according to manufacturer's protocol. For specific primers and oligo dT_AGC_ incubation temperatures were 50°C and 40°C, respectively.

PCR was conducted using different combinations of primers (listed in **Table S5**) and recombinant *Taq* DNA polymerase (Fermentas) using 30–40 amplification cycles and a 10 µl final volume. PCR products were treated with Klenow polymerase, identified by restriction mapping, cloned into pBS SK^+^ vector *Eco*RV site and sequenced. PCR amplifications of human cDNAs derived from the multiple tissue cDNA panel provided by P. Pruunsild [74], was carried out using recombinant Taq DNA polymerase and 40 cycles (**Figure S2**). DNA-free total RNAs from human tissues were purchased from BioChain and used in experiment shown in **Figure 2**. Endogenous NCAM1 transcripts were amplified with the following primer pairs: 6/7 ex Dir - 11 ex Rev primers, 6/7 ex Dir - 8 int Rev, 6/7 ex Dir - 9 int Rev A and 6/7 ex Dir - 9 int Rev B (nested PCR, 10 cycles). Endogenous *TXNDC12* and *KTI12* transcripts were detected with the following primer pairs: 2 ex Dir - 7 ex Rev, KTI Dir 1 - KTI Rev RT, 2 Ex Dir - EST1 Rev and 2 Ex Dir - EST2 Rev for nested PCR (30 cycles). The primers used for the detection of host gene, nested gene, potential TI-specific transcripts induced by nested genes and *PPIA* (peptidylprolyl isomerase A) are shown in **Table S5**.

### Ribonuclease protection assay

Equal amounts of total RNAs isolated after transfection were dissolved in FA hybridization cocktail and hybridized with different ^32^P-labeled riboprobes as described in [31]. Riboprobes were synthesized with T3 or T7 RNA polymerase (Fermentas) from appropriate recombinant pBS SK^+^ plasmids linearized with different restriction enzymes. Hybridization was carried out in a 8–10 µl volume. Single-stranded RNAs were removed with RNase A (40 µg/ml) and T1 (100 u/ml) (Fermentas) at 30°C (or 40°C for polyA probes) for 1 hour. RNases were removed with proteinase K (Fermentas) (200 µg/ml) in the presence of 0.5% sodium dodecyl sulfate (SDS) at 37°C for 1 hour. After phenol treatment dsRNAs were precipitated with ethanol in the presence of carrier tRNA (3 µg) and were dissolved in gel loading buffer containing 95% formamide. Protected ^32^P-labelled RNA fragments were analyzed on a 5% polyacrylamide gel and were detected by Personal FX phosphoimager (BioRad). Quantitation of the bands corresponding to transcripts was carried out by comparing their intensities to the reference, i.e. two-fold serial dilutions of the probe, and if necessary, size normalization was made. All RPA experiments were reproduced using total RNAs obtained from at least two separate transfections.

## Supporting Information

Figure S1 **TI effects induced by L1 5′ UTR and ORFs.** Quantitative detection of various minigene transcripts by RPA. Riborobes Fl_1-23-3_ and Int_1-23_ (lanes 1 and 11) are schematically shown above each panel. (A) Different types of 5′ UTR (Fl #11AS, RP, PA3), deletion (Δ) and mutation (M) constructs and (B) RP 5′ UTR+ORFs +/−, PA3 5′ UTR+ORFs and ORFs +/− used in transfection experiments are shown on top of each lane. Their structures are mapped to the schemes above panels. Protected transcripts (marked with arrows) are schematically shown by boxes and their sizes are given in nucleotides. Dashed lines/boxes show the remaining exon(s) not protected by the riboprobe used. In the case of RP 5′ UTR + ORFs (+/−) very faint signals were detected in the original autoradiogram. TE – transfection simulated with buffer.
(TIF)

Figure S2 **L1-induced TI determined from** ***NCAM1*** **endogenous transcripts.** (A) Detection of alternatively spliced *NCAM1* transcripts in different human cell lines using RT-PCR. (B) Detection of endogenous *NCAM1* transcripts in neuroblastoma cell line (Kelly) by RPA. Various transcripts (structures shown on the right) were detected with three different riboprobes shown above panels. Their abundance was determined from the comparison to 2-fold serial dilution of riboprobe (bottom right) after size normalization. (C) Genomic structures of the human and mouse *NCAM1* containing exons 7–1. Hatched boxes show intronic regions observed in RT-PCR. (D) Detection of *NCAM1* transcripts in mouse and human neuroblastoma cell lines and brain cells. Separation of the RT-PCR products with and without exon 9 are shown at the bottom of panels. Various alternatively spliced transcripts in all panels (mouse/human structures, sizes in nucleotides shown on right) were detected with primers specific to exons 7 and 11 and introns 8 and 9. (E) Analysis of the *NCAM1* transcripts containing exons 7–11. Endogenous transcripts (Ex-exon and Int-intron) shown on the right of panels were detected by RT-PCR. Intron-containing transcripts were further amplified by nested PCR. Note that *NCAM1* primary PCR product (Ex 7–9-Int, upper band) is also visible in some lanes.
(DOC)

Figure S3 **TI dependence on the location of L1 (A) or nested gene (B) relative to the intron retention and exonization effects in their upstream region.** Data analysis from Tables S1 and Table S3 and summary in Figure 7.
(TIF)

Figure S4 **TI determined from endogenous** ***NCAM1*** **transcripts derived from different human cell lines and tissues.**
(DOC)

Table S1 **Prediction of TI between human genes and intronic L1 retrotransposons.**
(DOC)

Table S2 **Distribution of additional aberrant transcripts in human genes containing intronic L1 retrotransposons.**
(DOC)

Table S3 **TI between host and nested genes.**
(DOC)

Table S4 **Search for additional aberrant transcripts in selected host genes shown in Table S2.**
(DOC)

Table S5 **Primers used in RT-PCR and construction of minigenes.**
(XLS)
