# Supplementary figures and images for: Intronic L1 Retrotransposons and Nested Genes Cause Transcriptional Interference by Inducing Intron Retention, Exonization and Cryptic Polyadenylation
Source: PLoS One. 2011 Oct 13;6(10):e26099. doi: 10.1371/journal.pone.0026099 (PMC3192792; doi:10.1371/journal.pone.0026099)

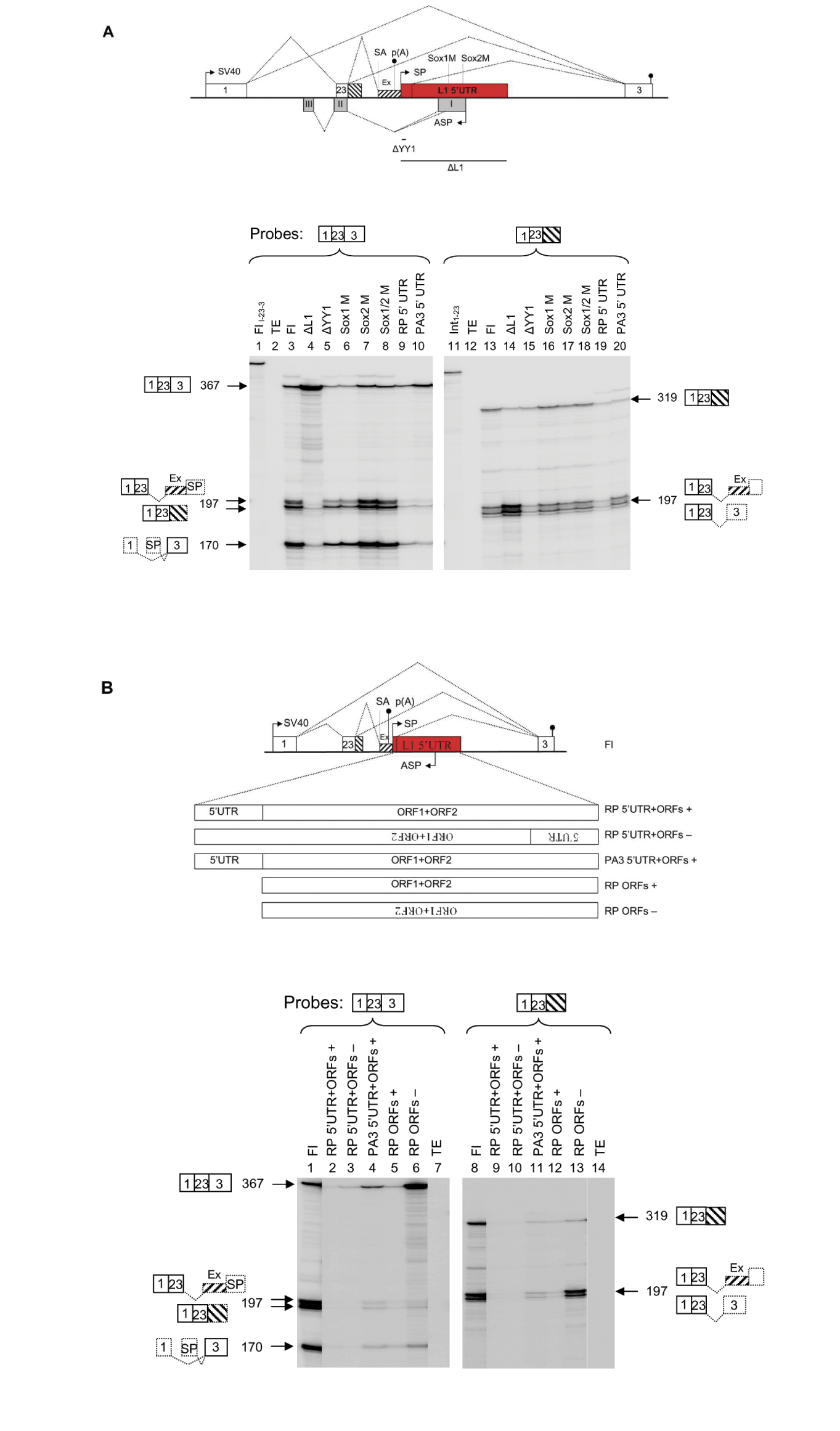

Supplement: Figure S1 — TI effects induced by L1 5′ UTR and ORFs. Quantitative detection of various minigene transcripts by RPA. Riborobes Fl1-23-3 and Int1-23 (lanes 1 and 11) are schematically shown above each panel. (A) Different types of 5′ UTR (Fl #11AS, RP, PA3), deletion (Δ) and mutation (M) constructs and (B) RP 5′ UTR+ORFs +/−, PA3 5′ UTR+ORFs and ORFs +/− used in transfection experiments are shown on top of each lane. Their structures are mapped to the schemes above panels. Protected transcripts (marked with arrows) are schematically shown by boxes and their sizes are given in nucleotides. Dashed lines/boxes show the remaining exon(s) not protected by the riboprobe used. In the case of RP 5′ UTR + ORFs (+/−) very faint signals were detected in the original autoradiogram. TE – transfection simulated with buffer. (TIF) [file pone.0026099.s001.tif]

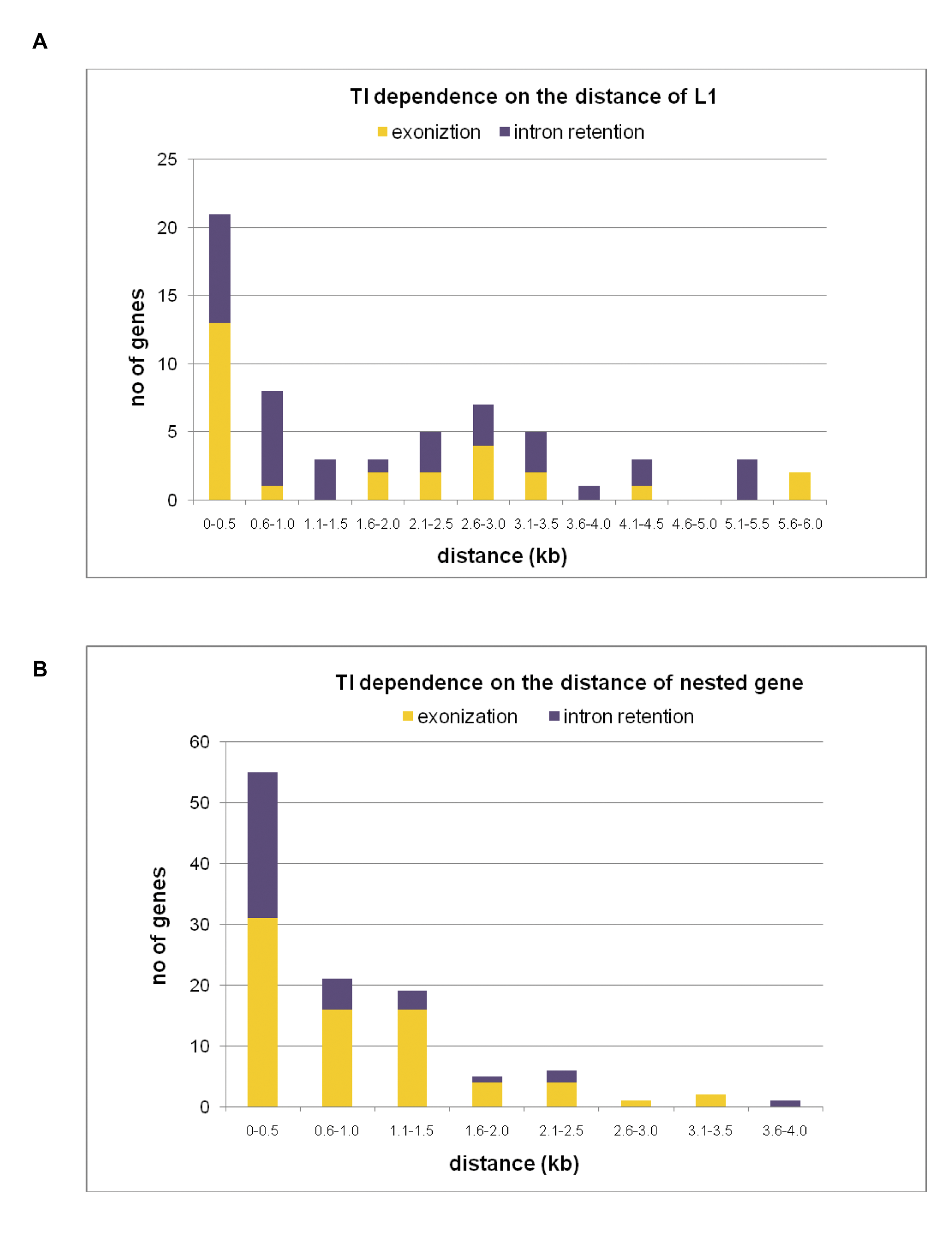

Supplement: Figure S3 — TI dependence on the location of L1 (A) or nested gene (B) relative to the intron retention and exonization effects in their upstream region. Data analysis from Tables S1 and Table S3 and summary in Figure 7. (TIF) [file pone.0026099.s003.tif]
