# Supplementary material for: Intronic L1 Retrotransposons and Nested Genes Cause Transcriptional Interference by Inducing Intron Retention, Exonization and Cryptic Polyadenylation
Source: PLoS One. 2011 Oct 13;6(10):e26099. doi: 10.1371/journal.pone.0026099 (PMC3192792; doi:10.1371/journal.pone.0026099)
Supplement: Figure S2 — L1-induced TI determined from NCAM1 endogenous transcripts. (A) Detection of alternatively spliced NCAM1 transcripts in different human cell lines using RT-PCR. (B) Detection of endogenous NCAM1 transcripts in neuroblastoma cell line (Kelly) by RPA. Various transcripts (structures shown on the right) were detected with three different riboprobes shown above panels. Their abundance was determined from the comparison to 2-fold serial dilution of riboprobe (bottom right) after size normalization. (C) Genomic structures of the human and mouse NCAM1 containing exons 7–1. Hatched boxes show intronic regions observed in RT-PCR. (D) Detection of NCAM1 transcripts in mouse and human neuroblastoma cell lines and brain cells. Separation of the RT-PCR products with and without exon 9 are shown at the bottom of panels. Various alternatively spliced transcripts in all panels (mouse/human structures, sizes in nucleotides shown on right) were detected with primers specific to exons 7 and 11 and introns 8 and 9. (E) Analysis of the NCAM1 transcripts containing exons 7–11. Endogenous transcripts (Ex-exon and Int-intron) shown on the right of panels were detected by RT-PCR. Intron-containing transcripts were further amplified by nested PCR. Note that NCAM1 primary PCR product (Ex 7–9-Int, upper band) is also visible in some lanes. (DOC) [file pone.0026099.s002.doc]

**
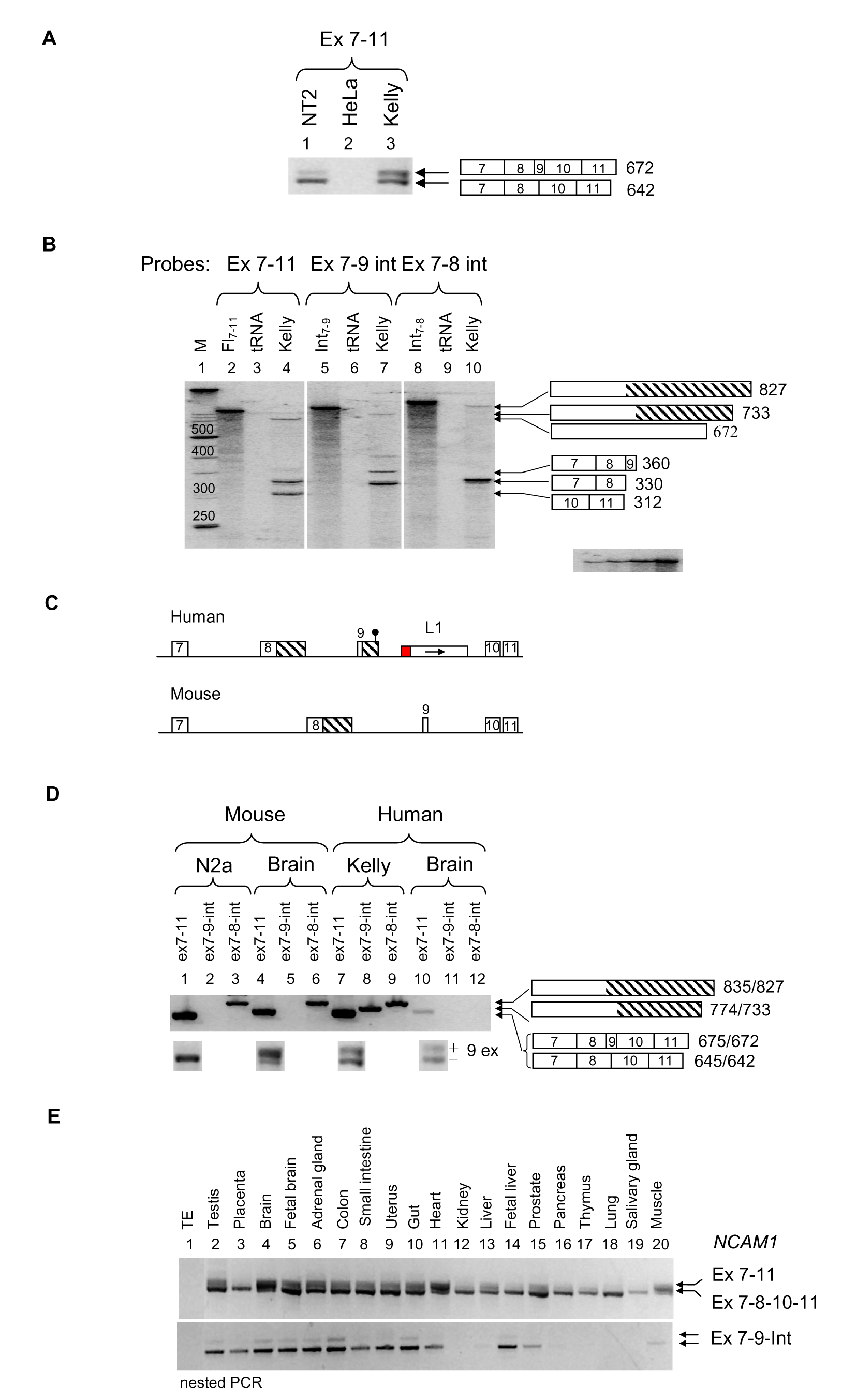
**

**Figure S2. TI determined from endogenous *NCAM1* transcripts derived from different human cell lines and tissues**

To reveal the potential of L1-induced TI *in vivo*, we analyzed *NCAM1* transcripts derived from different cell lines and tissues. Of the three cell lines tested, Fl and alternatively spliced transcripts (exon 9 skipping) were detected in teratocarcinoma and neuroblastoma cell lines (**panel A**). Quantitative analysis of *NCAM1* transcripts (**panel B**) derived from neuroblastoma cell line revealed intron 9 retention in about 15 % of transcripts, compared to Fl transcripts (lane 7). Intron 8 retention was observed in about 5 % of transcripts (lane 10). Exon 9 skipping was detected for most (about 80 %) transcripts (lane 4). Similar results, but with much weaker signals, were obtained for teratocarcinoma cell line (data not shown). These results suggest that L1 probably interferes with *NCAM1* transcription by causing intron retention to otherwise spliced exons.

To prove that intron 9 retention is specific to human *NCAM1* and does not occur, for instance, in mouse orthologous gene, which has no L1 in its intron 9 (**panel C**), we compared *NCAM1* transcripts derived from mouse and human cells. *NCAM1* Fl transcripts were detected in mouse and human brain cells and human, but not in mouse, neuroblastoma cell lines (**panel D**). Weak signal observed for human brain suggested a rather low level of transcription. In contrast, mouse brain had much higher level of transcription and also showed higher proportion of Fl transcripts (**panel D**, cf. lanes 4 and 10), however no intron 9-containing transcripts were detected in these cells (lane 5). In addition, transcripts with retained intron 8 were observed in both mouse and human cell lines and in mouse brain possibly indicating slow transcriptional elongation in intron 8 (necessary for inclusion of exon 9), as explained in ref [1]. Since very low level of *NCAM1* transcription was detected in the human brain, we decided to use nested-PCR in order to increase the sensitivity of detection. Also, to expand our analysis, we used a panel of cDNAs derived from different human tissues. **Panel E** shows that Fl and alternatively spliced *NCAM1* transcripts are present at variable amounts in most tissues analyzed (upper panel). From this experiment, intron 9 retention was detected in at least half of tissues (lower panel) and correlated well with the presence of exon 9 in these transcripts (e.g., testis, placenta, brain, etc., lanes 1-10). It is important to note that the L1 activity was previously detected in tissues, such as testis, placenta, brain and different embryonal tissues [2-4]. Therefore, it can be concluded that the presence of L1 in intron 9 of human *NCAM1* could possibly cause TI in multiple human tissues.

1. de la Mata M, Alonso CR, Kadener S, Fededa JP, Blaustein M, Pelisch F, Cramer P, Bentley D, Kornblihtt AR: **A slow RNA polymerase II affects alternative splicing in vivo**. *Mol Cell* 2003, **12**(2):525-532.

2. Mätlik K, Redik K, Speek M: **L1 antisense promoter drives tissue-specific transcription of human genes**. *J Biomed Biotechnol* 2006, **2006**(1):71753.

3. Ergun S, Buschmann C, Heukeshoven J, Dammann K, Schnieders F, Lauke H, Chalajour F, Kilic N, Stratling WH, Schumann GG: **Cell type-specific expression of LINE-1 open reading frames 1 and 2 in fetal and adult human tissues**. *J Biol Chem* 2004, **279**(26):27753-27763.

4. Coufal NG, Garcia-Perez JL, Peng GE, Yeo GW, Mu Y, Lovci MT, Morell M, O'Shea KS, Moran JV, Gage FH: **L1 retrotransposition in human neural progenitor cells**. *Nature* 2009, **460**(7259):1127-1131.
