# Supplementary material for: Intronic L1 Retrotransposons and Nested Genes Cause Transcriptional Interference by Inducing Intron Retention, Exonization and Cryptic Polyadenylation
Source: PLoS One. 2011 Oct 13;6(10):e26099. doi: 10.1371/journal.pone.0026099 (PMC3192792; doi:10.1371/journal.pone.0026099)
Supplement: Figure S4 — TI determined from endogenous NCAM1 transcripts derived from different human cell lines and tissues. (DOC) [file pone.0026099.s004.doc]

**TI determined from endogenous *NCAM1* transcripts derived from different human cell lines and tissues**

To reveal the potential of L1-induced TI *in vivo*, we analyzed *NCAM1* transcripts derived from different cell lines and tissues. Of the three cell lines tested, Fl and alternatively spliced transcripts (exon 9 skipping) were detected in teratocarcinoma and neuroblastoma cell lines (**panel A**). Quantitative analysis of *NCAM1* transcripts (**panel B**) derived from neuroblastoma cell line revealed intron 9 retention in about 15 % of transcripts, compared to Fl transcripts (lane 7). Intron 8 retention was observed in about 5 % of transcripts (lane 10). Exon 9 skipping was detected for most (about 80 %) transcripts (lane 4). Similar results, but with much weaker signals, were obtained for teratocarcinoma cell line (data not shown). These results suggest that L1 probably interferes with *NCAM1* transcription by causing intron retention to otherwise spliced exons.

To prove that intron 9 retention is specific to human *NCAM1* and does not occur, for instance, in mouse orthologous gene, which has no L1 in its intron 9 (**panel C**), we compared *NCAM1* transcripts derived from mouse and human cells. *NCAM1* Fl transcripts were detected in mouse and human brain cells and human, but not in mouse, neuroblastoma cell lines (**panel D**). Weak signal observed for human brain suggested a rather low level of transcription. In contrast, mouse brain had much higher level of transcription and also showed higher proportion of Fl transcripts (**panel D**, cf. lanes 4 and 10), however no intron 9-containing transcripts were detected in these cells (lane 5). In addition, transcripts with retained intron 8 were observed in both mouse and human cell lines and in mouse brain possibly indicating slow transcriptional elongation in intron 8 (necessary for inclusion of exon 9), as explained in ref [1]. Since very low level of *NCAM1* transcription was detected in the human brain, we decided to use nested-PCR in order to increase the sensitivity of detection. Also, to expand our analysis, we used a panel of cDNAs derived from different human tissues. **Panel E** shows that Fl and alternatively spliced *NCAM1* transcripts are present at variable amounts in most tissues analyzed (upper panel). From this experiment, intron 9 retention was detected in at least half of tissues (lower panel) and correlated well with the presence of exon 9 in these transcripts (e.g., testis, placenta, brain, etc., lanes 1-10). It is important to note that the L1 activity was previously detected in tissues, such as testis, placenta, brain and different embryonal tissues [2-4]. Therefore, it can be concluded that the presence of L1 in intron 9 of human *NCAM1* could possibly cause TI in multiple human tissues.

1. de la Mata M, Alonso CR, Kadener S, Fededa JP, Blaustein M, Pelisch F, Cramer P, Bentley D, Kornblihtt AR: **A slow RNA polymerase II affects alternative splicing in vivo**. *Mol Cell* 2003, **12**(2):525-532.

2. Mätlik K, Redik K, Speek M: **L1 antisense promoter drives tissue-specific transcription of human genes**. *J Biomed Biotechnol* 2006, **2006**(1):71753.

3. Ergun S, Buschmann C, Heukeshoven J, Dammann K, Schnieders F, Lauke H, Chalajour F, Kilic N, Stratling WH, Schumann GG: **Cell type-specific expression of LINE-1 open reading frames 1 and 2 in fetal and adult human tissues**. *J Biol Chem* 2004, **279**(26):27753-27763.

4. Coufal NG, Garcia-Perez JL, Peng GE, Yeo GW, Mu Y, Lovci MT, Morell M, O'Shea KS, Moran JV, Gage FH: **L1 retrotransposition in human neural progenitor cells**. *Nature* 2009, **460**(7259):1127-1131.
