# Supplementary material for: Intronic L1 Retrotransposons and Nested Genes Cause Transcriptional Interference by Inducing Intron Retention, Exonization and Cryptic Polyadenylation
Source: PLoS One. 2011 Oct 13;6(10):e26099. doi: 10.1371/journal.pone.0026099 (PMC3192792; doi:10.1371/journal.pone.0026099)
Supplement: Table S1 — Prediction of TI between human genes and intronic L1 retrotransposons. (DOC) [file pone.0026099.s005.doc]

Table S1. Prediction of TI between human genes and intronic L1 retrotransposons

| **No** | **UCSC Genome Browser** | **Host gene** | **Number of exons** | **L1**  **subfamily^a^** | **Location in intron** | **TI EST^b^** | **Expression^c^** | **Effects** |
| --- | --- | --- | --- | --- | --- | --- | --- | --- |
| 1 | chr1:108,478,501-108,545,500 | *SLC25A24* | 10 | PA3 | 3 | BF979584 | testis | exonization ~3.5 kb upstream |
| 2 | chr1:117,253,501-117,335,500 | *PTGFRN* | 9 | PA5 | 7 | BF063322 | colon tumor | intron retention ~0.2 kb upstream |
| 3 | chr1:170,485,919-170,577,251 | *DNM3* | 17 | PA3 | 15 | AK308992 | hippocampus | intron retention ~4.5 kb upstream |
| 4 | chr1:170,655,001-170,705,000 | *C1orf105* | 7 | PA6 | 1 | AI149902 | testis | exonization ~0.5 kb upstream and polyadenylation |
| 5 | chr1:171,844,001-171,906,500 | *ANKRD45* | 6 | PA6 | 2 | BC094694 | testis | intron retention ~1.4 kb upstream |
| 6 | chr1:171,948,501-172,026,500 | *KLHL20* | 12 | PA4 | 2 | DA467238 | cord blood | exonization ~2.7 kb upstream |
| 7 | chr2:24,150,442-24,250,124 | *LOC375190* | 11 | PA6 | 3 | DB500711  DA785655  DA808546 etc | hypothalamus  fetal brain  fetal brain | exonization 17 b upstream, L1 5’ UTR inclusion  exonization 17 b upstream, L1 5’ UTR inclusion  exonization 17 b upstream, L1 5’ UTR inclusion |
| 8 | chr2:48,767,417-48,836,384 | *LHCGR* | 11 | PA6 | 8 | BX097897  AI126141 | testis  testis | intron retention ~0.6 kb upstream and polyadenylation  intron retention ~0.6 kb upstream and polyadenylation |
| 9 | chr3:21,563,000-21,662,000 | *ZNF385D* | 9 | PA7 | 3 | BP276600  BG194196  BG195414 etc | kidney  fibrosarcoma cell line  fibrosarcoma cell line | exonization ~0.5 kb upstream  exonization ~0.5 kb upstream and polyadenylation  exonization ~0.5 kb upstream and polyadenylation |
| 10 | chr3:29,933,450-30,032,450 | *RBMS3* | 15 | PA4 | 14 | BU570213  AW779062  AF023259 etc | testis  pooled: fetal lung, testis, B-cell  fibroblast | intron retention ~2.2 kb upstream  intron retention ~2.5 kb upstream  intron retention ~2.6 kb upstream |
| 11 | chr3:38,389,425-38,433,425 | *XYLB* | 19 | PA3 | 18 | AB015046 | liver | intron retention ~4.1 kb upstream |
| 12 | chr4:86,102,034-86,154,820 | *C4ORF12* | 4 | PA4 | 2 | CK903613 | pancreas islets of langerhans | exonization ~2.2 kb upstream |
| 13 | chr5:74,023,652-74,049,706 | *HEXB* | 14 | PA3 | 6 | CR605235  CR595990  BX439122 etc | placenta  placenta  placenta | intron retention ~0.1 kb upstream  intron retention ~0.1 kb upstream  intron retention ~0.9 kb upstream |
| 14 | chr5:90,123,247-90,127,054 | *GPR98* | 90 | PA7 | 71 | CN430304 | embryonic stem cells | exonization ~1.9 kb upstream |
| 15 | chr5:126,257,213-126,266,024 | *MARCH3* | 5 | PA3 | 3 | DB520688  CA306167 | testis  alveolar macrophage | exonization ~2.8 kb upstream and polyadenylation  exonization ~2.8 kb upstream and polyadenylation |
| 16 | chr5:126,270,759-126,280,549 | *MARCH3* | 5 | PA3 | 3 | BC146948  BC146964  CV815936 | pooled from different tissues  pooled from different tissues  pluripotent cell line | intron retention ~5.1 kb upstream  intron retention ~5.1 kb upstream  intron retention 5.0 kb upstream |
| 17 | chr5:133,693,522-133,707,467 | *CDKL3* | 13 | PA6 | 4 | BQ948251 | epidermoid carcinoma, cell line | exonization ~2.7 kb upstream |
| 18 | chr6:46,275,001-46,375,000 | *RCAN2* | 5 | PA5 | 4 | BP199391 | brain | exonization ~2.3 kb upstream |
| 19 | chr6:54,278,335-54,311,667 | *TINAG* | 11 | PA7 | 4 | AW139886  AI393248  BI765216 etc | kidney  B-cell, chronic lymphotic leukemia  pooled colon, kidney, stomach | intron retention ~0.2 kb upstream and polyadenylation  intron retention ~0.2 kb upstream  exonization 12 b upstream, L1 5’ UTR inclusion |
| 20 | chr6:125,118,085-125,217,085 | *TCBA1* | 7 | PA3 | 5 | DA388857  DA493142 | thalamus  brain | exonization 41 b upstream, L1 5’UTR inclusion  exonization 41 b upstream |
| 21 | chr7:92,808,554-92,809,791 | *CCDC132* | 28 | PA3 | 23 | DB035845 | testis | exonization ~0.2 kb upstream |
| 22 | chr7:99,091,251-99,098,797 | *CYP3A5* | 14 | PA4 | 14 | BX648856  L26985  AW007836 etc | human small intestine  liver  colon | exonization ~0.2 kb upstream  intron retention 43 b upstream  intron retention 33 b upstream |
| 23 | chr7:102,206,708-102,210,020 | *MGC35361* | 8 | PA4 | 6 | BE778155 | retinoblastoma | exonization ~1.0 kb upstream |
| 24 | chr8:99,643,529-99,710,189 | *STK3* | 15 | PA3 | 11 | AK075229 | placenta | intron retention ~5.3 kb upstream |
| 25 | chr8:106,858,755-106,892,085 | *ZFPM2* | 8 | PA3 | 6 | AL699673  CB146270  BE866068 | n/a  liver HLK-3 cell line  bladder carcinoma cell line | intron retention ~2.9 kb upstream  exonization ~2.0 kb upstream  exonization ~2.0 kb upstream |
| 26 | chr9:72,009,193-72,025,433 | *AK124136* | 4 | PA5 | 3 | CR936772  BC040840 | endometrium carcinoma cell line  brain, hypothalamus | exonization ~0.4 kb upstream, L1 inclusion  intron retention ~3.4 kb upstream and polyadenylation |
| 27 | chr9:84,858,858-84,861,857 | *RASEF* | 17 | HS | 1 | AK056176  C75455  C75612 | NTera2D1 cell line  n/a  n/a | exonization ~0.1 kb upstream, L1 5’ UTR inclusion  exonization ~0.1 kb upstream, L1 5’ UTR inclusion  exonization ~0.1 kb upstream |
| 28 | chr9:112,585,924-112,596,750 | *MUSK* | 14 | HS | 13 | BG209775 | HT1080 cell line | intron retention ~3.1 kb upstream |
| 29 | chr10:31,767,997-31,817,998 | *ZEB1* | 13 | PA3 | 6 | AV741077 | cord blood | exonization ~2.7 kb upstream |
| 30 | chr10:95,264,301-95,280,967 | *CEP55* | 9 | PA4 | 8 | CA418379 | bone chondrosarcoma cell line | intron retention ~0.7 kb upstream |
| 31 | chr11:11,819,546-11,937,448 | *USP47* | 28 | PA10 | 4 | AW021906  BI492834 | fetal cochlea  fetal cochlea | intron retention ~0.6 kb upstream  intron retention ~0.5 kb upstream and polyadenylation |
| 32 | chr11:14,621,907-14,848,926 | *PDE3B* | 16 | PA5 | 2 | AB209326 | brain | intron retention ~1.0 kb upstream |
| 33 | chr11:112,337,205-112,654,368 | *NCAM1* | 19 | PA5 | 9 | BC029119  BX432004 | brain  fetal liver | intron retention ~2.0 kb upstream and polyadenylation  intron retention ~2.2 kb upstream |
| 34 | chr11:59,953,638-59,971,839 | *MS4A5* | 5 | PA2 | 4 | BU160183  DB029317 | testis  testis | exonization 11 b upstream, L1 5’ UTR inclusion  exonization 11 b upstream, L1 5’ UTR inclusion |
| 35 | chr12:44,867,155-44,883,819 | *SLC38A1* | 17 | PA6 | 16 | AK309410  DB279830  DB279836 | fetal brain  uterus  uterus | exonization ~0.2 kb upstream, L1 5’ UTR inclusion  intron retention ~1.3 kb upstream  intron retention ~1.3 kb upstream |
| 36 | chr12:60,839,067-60,938,912 | *FAM19A2* | 6 | PA8A | 1 | DB092379 | testis | intron retention ~0.7 kb upstream |
| 37 | chr12:94,783,900-94,862,645 | *CCDC38* | 16 | PA3 | 2 | BC047659  BI459337 | testis  testis | exonization ~5.9 kb upstream and polyadenylation  exonization ~5.9 kb upstream |
| 38 | chr13:47,928,551-47,945,050 | *RB1* | 27 | HS | 23 | AI745643  AI745644 | ovarian tumor, pooled 5  ovarian tumor, pooled 5 | intron retention ~0.1 kb upstream  intron retention ~0.1 kb upstream |
| 39 | chr13:48,162,301-48,184,300 | *CYSLTR2* | 6 | PA5 | 5 | DA978269 | synovial membrane tissue from rheumatioid arthritis | intron retention within L1 5’ UTR |
| 40 | chr14:57,994,414-58,093,713 | *KIAA0586* | 32 | PA7 | 30 | AW853482 | colon | exonization ~4.3 kb upstream |
| 41 | chr14:80,635,366-80,657,431 | *TSHR* | 10 | PA3 | 8 | BC024205  BE206608  BC120972 etc | ovary, adenocarcinoma  ovarian adenocarcinoma cell line  n/a | exonization ~5.6 kb upstream and polyadenylation  exonization ~5.6 kb upstream  intron retention ~5.3 kb upstream |
| 42 | chr15:49,856,575-49,873,238 | *TMOD2* | 11 | PA7 | 8 | BC052749  BE089837  BE089838 | ovary, pooled from 3 adults  breast  breast | exonization ~3.4 kb upstream and polyadenylation  intron retention ~3.9 kb upstream  intron retention ~3.7 kb upstream |
| 43 | chr15:54,443,120-54,542,228 | *TEX9* | 13 | PA6 | 9 | AL832050  CR621164 | bone marrow  Jurkat cell line | intron retention ~0.6 kb upstream and polyadenylation  intron retention ~2.7 kb upstream |
| 44 | chr15:75,186,510-75,285,618 | SGK269 | 6 | PA4 | 4 | AK074157 | spleen | intron retention within L1 5’ UTR |
| 45 | chr18:31,412,385-31,511,393 | *GALNT1* | 12 | PA5 | 1 | AI222691 | kidney | exonization ~0.2 kb upstream |
| 46 | chr19:11,827,501-11,927,500 | *ZNF69* | 5 | PA5 | 1 | BG393583 | testis embryonal carcinoma cell line | intron retention ~1.3 kb upstream |
| 47 | chr19:45,223,890-45,257,222 | *ZNF780B* | 5 | PA4 | 4 | AW803563 | uterus | intron retention ~3.2 kb upstream |
| 48 | chr20:25,115,451-25,115,753 | *AX747658* | 6 | PA6 | 4/5 | DA176713 | amygdala | exonization 16 b upstream, L1 5’ UTR inclusion |
| 49 | chr20:33,120,279-33,136,613 | *TRPC4AP* | 19 | PA5 | 2 | DA794245  DB292589 | fetal brain  uterus | intron retention ~0.3 kb upstream  intron retention ~0.3 kb upstream |
| 50 | chrX:154,382,780-154,427,212 | *TMLHE* | 8 | HS | 3 | AW295048 | lung | intron retention ~2.2 kb upstream and polyadenylation |

^a^L1 subfamily [26].

^b^Transcriptional interference (TI) predicted from EST. Genbank accesion numbers, etc >3 ESTs.

^c^Expression of ESTs in different tissues (n/a, not available).
