# Supplementary material for: Intronic L1 Retrotransposons and Nested Genes Cause Transcriptional Interference by Inducing Intron Retention, Exonization and Cryptic Polyadenylation
Source: PLoS One. 2011 Oct 13;6(10):e26099. doi: 10.1371/journal.pone.0026099 (PMC3192792; doi:10.1371/journal.pone.0026099)
Supplement: Table S2 — Distribution of additional aberrant transcripts in human genes containing intronic L1 retrotransposons. (DOC) [file pone.0026099.s006.doc]

Table S2. Distribution of additional aberrant transcripts in human genes containing intronic L1 retrotransposons

| **No** | **UCSC Genome Browser** | **Host gene** | **Number of exons** | **Location of L1** | **Nested (pseudo)gene involved in TI**  **(?-unknown**  **– not detected)** | **Location of nested (pseudo)**  **gene** | **TI ESTs** | **Effect**  **(ex-exonization, int-intron**  **retention, polyA-polyadenylation)** |
| --- | --- | --- | --- | --- | --- | --- | --- | --- |
| 1 | chr1:108,478,501-108,545,500 | *SLC25A24* | 10 | intron 3 | SVA | intron 4 | BC017035 | ex within, polyA |
| 2 | chr1:117,253,501-117,335,500 | *PTGFRN* | 9 | intron 7 | L1MB7 | intron 6 | CR741095 | int, within |
| 3 | chr1:170,038,389-170,660,385 | *DNM3* | 17 | intron 15 | MIR | intron 12 | BC064546  AK056279  DB323831 | ex within  ex within  ex within |
| 4 | chr1:170,655,001-170,705,000 | *C1orf105* | 7 | intron 1 | DB058090 | intron 6 | AA923390 | ex ~0.2 kb upstream |
| 5 | chr1:171,844,001-171,906,500 | *ANKRD45* | 6 | intron 2 | – | – | – | – |
| 6 | chr1:171,948,501-172,026,500 | *KLHL20* | 12 | intron 2 | – | – | – | – |
| 7 | chr2:24,150,442-24,250,124 | *LOC375190* | 11 | intron 3 | – | – | – | – |
| 8 | chr2:48,767,417-48,836,384 | *LHCGR* | 11 | intron 8 | – | – | – | – |
| 9 | chr3:21,412,022-21,800,151 | *ZNF385D* | 9 | intron 3 | F13195 | intron 5 | DB518262  CA428944  DB511217 etc | int ~0.2 kb upstream  int ~0.2 kb upstream  int ~0.2 kb upstream |
| 10 | chr3:29,262,718-30,053,728 | *RBMS3* | 15 | intron 14 | CV325146  BG944978  AluSg  DW423659 | intron 2  intron 5  intron 11 | DA695573  BQ876639  BI769065  AA523313  BX281096 | int, within  ex ~0.6 kb upstream  int ~0.7 kb upstream  int ~0.8 kb upstream  ex ~0.1 kb upstream |
| 11 | chr3:38,360,224-38,432,225 | *XYLB* | 19 | intron 18 | BU733607  MIR | intron 2  intron 9 | BC039712  BI917458  BU959995  BE772384 | ex ~0.7 kb upstream, polyA  ex ~0.7 kb upstream  ex within  int ~0.3 kb upstream |
| 12 | chr4:86,102,034-86,154,820 | *C4ORF12* | 4 | intron 2 | – | – | – | – |
| 13 | chr5:74,013,882-74,054,348 | *HEXB* | 14 | intron 6 | AluSx  AluSx  N57757 | intron 2  intron 9  intron 13 | AU117719  BM473122  BI055063  BI916164 | int ~0.6 kb upstream  int ~0.6 kb upstream  int 55 b upstream  int 38 b upstream |
| 14 | chr5:89,871,284-90,505,950 | *GPR98* | 90 | intron 71 | AluJo  AluSc | intron 20  intron 59 | AB075823  AL136541 | int ~1.3 kb upstream  int within |
| 15 | chr5:126,217,549-126,403,888 | *MARCH3* | 5 | intron 3 | MIR | intron 2 | DR003379  DR003377 | ex ~1.6 kb upstream  ex ~1.6 kb upstream |
| 16 | chr5:133,653,108-133,735,089 | *CDKL3* | 13 | intron 4 | AluSx  BX951887 | intron 6  intron 12 | AV717506  AA868305 | ex within  int within |
| 17 | chr6:46,285,001-46,585,000 | *RCAN2* | 5 | intron 4 | – | – | – | – |
| 18 | chr6:54,280,503-54,365,501 | *TINAG* | 11 | intron 4 | BX107018  AluSq | intron 2  intron 3 | BG427401  AI245383  AI245871 etc  BC056235 | int within  int ~0.8 kb upstream  int ~0.8 kb upstream  int within, polyA |
| 19 | chr6:124,153,111-125,250,691 | *TCBA1* | 7 | intron 5 | BG198302  CN357777  DA104147 | intron 1  intron 3  intron 4 | AF150231  AI692199  AB070452 | ex within  ex within  int ~0.6 kb upstream |
| 20 | chr7:92,808,554-92,809,791 | *CCDC132* | 28 | intron 23 | AluJo  MER5C  Tigger1  L2  DB223772 | intron 4  intron 6  intron 11  intron 12  intron 27 | DB217907  DB149712  DR001484  AL832393  BC047757  BQ719613  BQ721562  AI793238  AI253087  BC017888  DB179460 | int ~0.4 kb upstream  int ~0.2 kb upstream  int within  ex ~0.4 kb upstream  int ~0.2 kb upstream, polyA  int ~0.6 kb upstream  int ~0.4 kb upstream  int ~0.4 kb upstream, polyA  int ~0.4 kb upstream  int ~0.4 kb upstream, polyA  int~ 1.0 kb upstream |
| 21 | chr7:99,083,045-99,173,917 | *CYP3A5* | 14 | intron 11 | MIRb  DA643057  Charlie12 (MER1)  L1MD  L1MB7  BX090630 | intron 1  intron 3  intron 5  intron 8  intron 12 | AF315325  AK097322  BX495304  BX495300 etc  BC026255  BC022298  AK299002 etc  BX483833  AV646642  AV646509 | ex ~1.6 kb upstream  int ~0.2 kb upstream  int ~0.2 kb upstream  int ~0.2 kb upstream  int within  int within, polyA  int within  int within  int ~0.4 kb upstream  int ~0.4 kb upstream |
| 22 | chr7:102,171,553-102,245,174 | *MGC35361* | 8 | intron 6 | BQ930668  L1PA16 | intron 2  intron 4 | BG576813  BE147694 | ex within  ex 8 b upstream, inclusion |
| 23 | chr8:99,530,053-100,036,476 | *STK3* | 15 | intron 11 | BF882212  L1PA5  L2 | intron 3  intron 3  intron 12 | CR627416  AL710499  DB444298 | int ~0.5 kb upstream, polyA  ex ~0.6 kb upstream  ex within |
| 24 | chr8:106,390,959-106,900,916 | *ZFPM2* | 8 | intron 6 | AluJo | intron 3 | BX394154 | ex ~0.6 kb upstream |
| 25 | chr9:71,995,387-72,068,471 | *AK124136* | 4 | intron 3 | – | – | – | – |
| 26 | chr9:84,779,358-84,880,607 | *RASEF* | 17 | intron 1 | – | – | – | – |
| 27 | chr9:112,464,663-112,610,827 | *MUSK* | 14 | intron 13 | AluSx  DB517750 | intron 5  intron 8 | AI302067  AI800924 | ex ~0.6 kb upstream  ex ~0.3 kb upstream |
| 28 | chr10:31,634,494-31,860,502 | *ZEB1* | 13 | intron 6 | AA496080 | intron 2 | DA239175 | ex 10 b upstream |
| 29 | chr10:95,245,967-95,279,300 | *CEP55* | 9 | intron 8 | AluSg  AluSg | intron 7  intron 8 | BU630927  CA418379 | int ~1.0 kb upstream, polyA  int within |
| 30 | chr11:11,819,546-11,937,448 | *USP47* | 28 | intron 4 | AluY  CV342185 | intron 12  intron 22 | CR749502  DA829585 | ex within, polyA  int ~0.2 kb upstream |
| 31 | chr11:14,621,907-14,848,926 | *PDE3B* | 16 | intron 2 | – | – | – | – |
| 32 | chr11:112,337,205-112,654,368 | *NCAM1* | 19 | intron 9 | BI041199 | intron 16 | DA406257 | int 2.0 kb upstream |
| 33 | chr11:59,953,638-59,971,839 | *MS4A5* | 5 | intron 4 | – | – | – | – |
| 34 | chr12:44,860,424-44,950,000 | *SLC38A1* | 17 | intron 16 | – | – | – | – |
| 35 | chr12:60,379,777-60,950,390 | *FAM19A2* | 6 | intron 1 | – | – | – | – |
| 36 | chr12:94,783,900-94,862,645 | *CCDC38* | 16 | intron 2 | MIRb | intron 7 | CR739944 | ex ~0.3 kb upstream |
| 37 | chr13:47,775,493-47,960,087 | *RB1* | 27 | intron 23 | CV349601 | intron 2 | BQ021681 | ex ~1.2 kb upstream, polyA |
| 38 | chr13:48,125,551-48,181,550 | *CYSLTR2* | 6 | intron 5 | – | – | – | – |
| 39 | chr14:57,963,414-58,086,713 | *KIAA0586* | 32 | intron 30 | DB344254  **?**  M86051  CA392494 | intron 2  intron 25  intron 28  intron 31 | DC402914  AK302718  AK310356  AK302579  AI223212 | ex within  int  ex ~0.4 kb upstream  ex ~0.4 kb upstream  ex ~0.4 kb upstream |
| 40 | chr14:80,487,525-80,686,118 | *TSHR* | 10 | intron 8 | MIRb  MLT1G | intron 3  intron 8 | DB121247  BE158931 | int ~0.3 kb upstream  ex ~0.2 kb upstream |
| 41 | chr15:49,829,911-49,892,502 | *TMOD2* | 11 | intron 8 | AW960153 | intron 2 | BM811468 | int within |
| 42 | chr15:54,443,120-54,526,228 | *TEX9* | 13 | intron 9 | CR737334 | intron 4 | AA020919 | ex within |
| 43 | chr15:75,183,996-75,367,991 | SGK269 | 6 | intron 4 | – | – | – | – |
| 44 | chr18:31,412,801-31,561,300 | *GALNT1* | 12 | intron 1 | L1ME3 | intron3 | BC047746  BQ716608 | int within, polyA  int within |
| 45 | chr19:11,858,501-11,888,500 | *ZNF69* | 5 | intron 4 | – | – | – | – |
| 46 | chr19:45,223,890-45,257,222 | *ZNF780B* | 5 | intron 4 | – | – | – | – |
| 47 | chr20:25,112,008-25,126,754 | *AX747658* | 6 | intron 4/5 | – | – | – | – |
| 48 | chr20:33,047,589-33,145,950 | *TRPC4AP* | 19 | intron 2 | Charlie8  BX471622 | intron 11  intron 13 | BI917766  DB137429 | ex ~0.3 kb upstream  int 42 b upstream |
| 49 | chrX:154,370,005-154,498,303 | *TMLHE* | 8 | intron 3 | BG697094 | intron 6 | AK310667 | int ~0.1 kb upstream |
