# Supplementary material for: Intronic L1 Retrotransposons and Nested Genes Cause Transcriptional Interference by Inducing Intron Retention, Exonization and Cryptic Polyadenylation
Source: PLoS One. 2011 Oct 13;6(10):e26099. doi: 10.1371/journal.pone.0026099 (PMC3192792; doi:10.1371/journal.pone.0026099)
Supplement: Table S3 — TI between host and nested genes. (DOC) [file pone.0026099.s007.doc]

Table S3. TI between host and nested genes

| ***No*** | ***UCSC Genome Browser*** | ***Host gene*** | ***Number of exons*** | ***Single exon***  ***nested gene***  ***(EST)*** | ***Coding (c)***  ***noncoding (nc)*** | ***Location***  ***in intron*** | ***Potential TI***  ***in mRNA/EST***  ***(etc >3 ESTs)*** | ***Expression***  ***(n/a not available)*** | ***Effect (ex-exonization, int-intron***  ***retention, polyA-polyadenylation)*** |
| --- | --- | --- | --- | --- | --- | --- | --- | --- | --- |
|  |  |  |  |  |  |  |  |  |  |
| 1 | chr1:608,396-618,272 | AK125248 | 6 | *OR4F16* | c | 3 | AW104412 | pooled: fetal lung, testis, B-cell | ex ~2.5 kb upstream |
| 2 | chr1:2,302,778-2,313,888 | *MORN1* | 14 | *AK055432* | c | 3  4 | AI701834  AI458216  BF446683 etc  BF437812  BP334250 | lung, carcinoid  lung, carcinoid  lung, carcinoid  prostate  salivary gland | int, within  int, within  int, within  int, within  int, within |
| 3 | chr1:2,290,418-2,307,084 | *MORN1* | 14 | AK128431 | nc | 6 | AK299217  BX104597  DB216072 etc | teratocarcinoma cell line  n/a  trachea | ex ~0.3 kb upstream, inclusion  ex ~0.3 kb upstream  ex ~0.3 kb upstream |
| 4 | chr1:2,270,063-2,279,939 | *MORN1* | 14 | BE243880 | nc | 10 | BC021704  BU741473  BX281965 etc | testis  fetal and adult eye  testis | ex ~0.4 kb upstream, polyA  ex ~0.4 kb upstream, polyA  ex ~0.4 kb upstream |
| 5 | chr1:3,738,890-3,761,111 | *KIAA0562* | 22 | AK091714 | nc | 6 | BC050721 | skin, melanotic melanoma | ex ~0.5 kb upstream, polyA |
|  |  |  |  |  |  |  | EL737107 | mixed | ex ~0.5 kb upstream |
| 6 | chr1:3,731,910-3,740,029 | *KIAA0562* | 22 | AA678361 | nc | 14 | BX097361 | fetal liver spleen | int, within |
| 7 | chr1:7,068,208-7,083,038 | *CAMTA1* | 23 | AK093159 | nc | 4 | DA404291 | thalamus | int ~1.5 kb upstream |
| 8 | chr1:9,554,659-9,562,073 | *SLC25A33* | 7 | *AK130277* | c | 5 | CN261786 | embryonic stem cells | int ~2.3 kb upstream |
| 9 | chr1:21,663,751-21,667,458 | *NBPF3* | 15 | AI378933 | nc | 2 | DB226908 | tachea | int ~0.2 kb upstream |
| 10 | chr1:52,075,586-52,079,293 | *NRD1* | 33 | AK090844 | nc | 2 | BM808991 | hippocampus | int ~0.5 kb upstream |
|  |  |  |  |  |  |  | BQ707371 | spleen | int ~0.5 kb upstream |
|  |  |  |  |  |  |  | AJ711255 etc | heart | int ~0.5 kb upstream |
| 11 | chr1:52,268,573-52,276,404 | *TXNDC12* | 7 | *KTI12* | c | 2 | DA735586 | teratocarcinoma cell line | ex ~0.3 kb upstream, inclusion |
|  |  |  |  |  |  |  | DA277685 | corpus callosum | ex ~0.3 kb upstream, inclusion |
|  |  |  |  |  |  |  | DA719333 | teratocarcinoma cell line | ex ~0.3 kb upstream, inclusion |
| 12 | chr1:71,120,738-71,124,445 | *PTGER3* | 5 | BX117888 | nc | 6 | U13216 | small intestine | ex ~0.3 kb upstream, inclusion |
|  |  |  |  |  |  |  | BC118659 | PCR rescued clones | ex ~0.3 kb upstream, inclusion |
|  |  |  |  |  |  |  | BC118578 | PCR rescued clones | ex ~0.3 kb upstream, inclusion |
| 13 | chr1:117,107,387-117,114,803 | *CD2* | 5 | AK127925 | nc | 4 | AK310676 | thymus | int, within |
|  |  |  |  |  |  |  | AK301154 | spleen | int, within |
|  |  |  |  |  |  |  | AK303367 | thymus | int, within |
| 14 | chr1:117,422,785-117,433,909 | *TTF2* | 23 | CR614106 | nc | 10 | BC030058 | brain, neuroblastoma | ex ~0.3 kb upstream, polyA |
| 15 | chr1:154,646,010-154,651,572 | *C1orf61* | 7 | *AK095622* | c | 4 | AK095626  BF526416  DA497813 etc | fetal brain  glioblastoma  fetal brain | ex ~1.5 kb upstream, inclusion  ex ~1.5 kb upstream  ex ~1.5 kb upstream |
| 16 | chr1:154,639,523-154,646,939 | *C1orf61* | 7 | *CR612522* | c | 5 | BG913317 | oligodendroglioma | int, within |
| 17 | chr1:160,096,836-160,107,946 | *ATF6* | 16 | DB370980 | nc | 14 | AB208929 | brain | int, within |
| 18 | chr1:163,720,170-163,731,280 | *AK093132* | 6 | AK125831 | nc | 4 | BC030596  BC033551  F28986 | testis  testis  pectoral muscle | ex ~1.2 kb upstream, polyA  ex ~3.5 kb upstream, polyA  ex ~3.5 kb upstream |
| 19 | chr1:170,694,820-170,708,886 | *C1orf105* | 7 | DB058090 | nc | 6 | AA923390 | mixed fetal lung, testis, B-cell | ex ~0.2 kb upstream |
| 20 | chr1:178,094,908-178,105,930 | *TOR1AIP2* | 6 | *IFRG15* | c | 2 | BX324877  AK289793  BX110121 etc | placenta  brain  melanotic melanoma | ex ~0.7 kb upstream, inclusion  ex ~0.7 kb upstream, inclusion  ex ~0.7 kb upstream |
| 21 | chr1:204,643,602-204,653,513 | *SRGAP2* | 20 | AK025818 | nc | 7 | BC112927  BM478763  BF571976 etc | mammary adenocarcinoma  skin melanotic melanoma  skin melanotic melanoma | int ~2.4 kb upstream, polyA  int ~2.4 kb upstream  int ~2.4 kb upstream |
| 22 | chr1:204,688,530-204,700,889 | *SRGAP2* | 20 | BQ232762 | nc | 17 | AK295845  AK293335  BE273609 | hippocampus  glioma  kidney, renal cell adenocarcinoma | ex ~0.2 kb upstream  ex ~0.2 kb upstream  ex ~0.2 kb upstream |
| 23 | chr1:231,572,834-231,580,256 | *KIAA1804* | 10 | AL698395 | nc | 6 | AJ311797  BF352800 | heart  head_neck | int, within  int, within |
| 24 | chr1:232,913,385-232,920,807 | *CR596412* | 4 | BQ648520 | nc | 2 | AK127956 | esophageal, tumor tissue | ex ~1.3 kb upstream, inclusion, polyA |
|  |  |  |  |  |  |  | DB013525 | esophageal, tumor tissue | ex ~1.3 kb upstream |
| 25 | chr1:239,856,826-239,871,671 | *OPN3* | 4 | *CHML* | c | 1 | DN996875 | breast cancer tissue | ex ~0.1 kb upstream, inclusion |
|  |  |  |  |  |  |  | DA323545 | hippocampus | ex ~0.1 kb upstream, inclusion |
|  |  |  |  |  |  |  | AA745052 | breast | ex ~0.1 kb upstream |
| 26 | chr1:241,438,727-241,443,706 | *CEP170* | 20 | BG035146 | nc | 4 | AK310003 | skeletal muscle | int ~1.4 kb upstream |
|  |  |  |  |  |  |  | DA899136 | skeletal muscle | int ~1.4 kb upstream |
| 27 | chr1:241,533,073-241,538,021 | *SDCCAG8* | 18 | CA772630 | nc | 7 | BX452685  BX452686 | adult brain  adult brain | int, within  int, within |
|  |  |  |  |  |  |  | AK023586  BU949558  AW513195 etc | placenta  pancreas  uterus, endometrial adenocarcinoma | ex ~0.3 kb upstream, inclusion  ex ~0.3 kb upstream, inclusion  ex ~0.3 kb upstream, inclusion |
| 28 | chr1:241,703,452-241,714,466 | *SDCCAG8* | 18 | AK024613 | nc | 16 | BU616964 | chondrosarcoma cell line | ex ~1.5 kb upstream, polyA |
|  |  |  |  |  |  |  | BM555911 | amelanotic melanoma, cell line | ex ~1.5 kb upstream, polyA |
|  |  |  |  |  |  |  | BM808076 | amelanotic melanoma, cell line | ex ~1.5 kb upstream, polyA |
| 29 | chr1:244,552,265-244,558,119 | *SMYD3* | 12 | AL134398 | nc | 5 | BX341831 | HeLa | int, within |
| 30 | chr1:244,552,265-244,558,119 | *SMYD3* | 12 | AW811233 | nc | 5 | BC107725 | brain, primitive neuroectodermal | ex ~0.7 kb upstream, polyA |
| 31 | chr1:247,171,869-247,181,691 | *PGBD2* | 3 | AK021482 | nc | 2 | DA629789 | kidney | int ~0.6 kb upstream |
| 32 | chr3:3,142,995-3,149,579 | *TRNT1* | 10 | TRNT1 | nc | 2 | BC005184  AK124137  CB052373 etc  DA041296 | bone marrow, leukemia  testis  soft tissue, leiomyosarcoma  bladder | ex ~0.1 kb upstream, inclusion, polyA  ex ~0.1 kb upstream, inclusion  ex ~0.1 kb upstream, inclusion  int ~0.9 kb upstream |
| 33 | chr3:21,447,991-21,457,872 | *ZNF385D* | 9 | F13195 | nc | 5 | DB518262  CA428944  DB511217 etc | testis  chondrosarcoma cell Line CS8  testis | int ~ 0.2 kb upstream  int ~ 0.2 kb upstream  int ~ 0.2 kb upstream |
| 34 | chr3:29,446,507-29,456,766 | *RBMS3* | 15 | CV325146 | nc | 2 | DA695573 | teratocarcinoma cell line | int, within |
| 35 | chr3:29,476,786-29,481,765 | *RBMS3* | 15 | BG944978 | nc | 2 | BQ876639 | sympathetic trunk | ex ~0.6 kb upstream |
| 36 | chr3:29,915,688-29,923,967 | *RBMS3* | 15 | DW423659 | nc | 11 | BX281096 | five pooled sarcomas | ex ~0.1 kb upstream |
| 37 | chr3:38,362,978-38,383,647 | *XYLB* | 19 | BU733607 | nc | 2 | BC03971  BI917458  BU959995 | fetal brain  brain  mixed | ex ~0.7 kb upstream, inclusion, polyA  ex ~0.7 kb upstream  ex, within |
| 38 | chr3:57,811,178-57,812,740 | *SLMAP* | 21 | AW082394 | nc | 5 | AI016499 | testis | ex ~0.2 kb upstream, inclusion |
| 39 | chr3:57,883,580-57,891,634 | *SLMAP* | 21 | BF820030 | nc | 20 | BI562293 | testis | int ~0.3 kb upstream |
| 40 | chr3:64,702,882-64,706,836 | BC040632 | 6 | AI208212 | nc | 1 | DA212367 | brain | ex ~0.5 kb upstream |
|  |  |  |  |  |  |  | CR743082 | testis | ex ~0.5 kb upstream |
| 41 | chr3:64,932,813-64,938,086 | BC040632 | 6 | BQ446624 | nc | 4 | AK057923 | kidney | ex ~1.1 kb upstream, inclusion |
| 42 | chr3:65,681,216-65,689,126 | *MAGI1* | 21 | BC014063 | nc | 1 | DA620154 | leukemia cell line | ex ~2.1 kb upstream |
| 43 | chr3:73,190,024-73,197,934 | *PPP4R2* | 9 | *FLJ10213* | c | 5 | BM921339 | pooled: brain, lung, testis | int ~0.6 kb upstream |
| 44 | chr3:79,640,829-79,648,739 | *ROBO1* | 30 | DN913944 | nc | 1 | AW184011 | pooled: fetal lung, testis, B-cell | ex ~1.2 kb upstream |
| 45 | chr3:100,897,132-100,913,886 | *ROBO1* | 30 | N42764 | nc | 3 | AK311449  DA738395  BF132395 | teratocarcinoma cell line  teratocarcinoma cell line  teratocarcinoma cell line | ex ~0.1 kb upstream  ex ~0.1 kb upstream  ex ~0.1 kb upstream |
| 46 | chr3:100,891,066-100,895,753 | *COL8A1* | 5 | BG993882 | nc | 3 | CN414930 | embryonic stem cells | int ~0.3 kb upstream |
| 47 | chr3:112,918,555-112,932,617 | *PLCXD2* | 5 | AX747104 | nc | 3 | PLCXD2 | bladder, mouth, placenta | ex ~3 kb upstream, inclusion |
|  |  |  |  |  |  |  | BX493293 | n/a | ex ~3 kb upstream |
| 48 | chr3:116,226,809-116,230,324 | *ZBTB20* | 11 | BE669590 | nc | 1 | AA464297 | ovarian tumor | ex ~1 kb upstream |
|  |  |  |  |  |  |  | DA984535 | synovium tissue from rheumatoid arthritis | ex ~1 kb upstream |
| 49 | chr3:115,859,295-115,861,073 | *ZBTB20* | 11 | AW293457 | nc | 5 | AI828036 | pooled: fetal lung, testis, B-cell | ex 5 nt upstream, inclusion |
| 50 | chr3:115,983,576-116,004,386 | *ZBTB20* | 11 | AA857492 | nc | 5 | BF064120 | fetal kidney | ex ~0.7 kb upstream |
| 51 | chr3:117,616,478-117,619,993 | *LSAMP* | 7 | DA125448 | nc | 1 | EF491802 | pooled: 18 normal tissues | ex ~1.6 kb upstream |
| 52 | chr3:117,040,992-117,046,265 | *LSAMP* | 7 | DA568784 | nc | 6 | AW379374 | head, neck | int ~0.9 kb upstream |
| 53 | chr3:120,212,916-120,216,431 | *IGSF11* | 9 | AW894892 | nc | 3 | AA292896 | testis | ex ~1.2 kb upstream |
| 54 | chr3:120,120,574-120,131,773 | *IGSF11* | 9 | BF061710 | nc | 6 | BQ424971 | skin, melanotic melanoma | int ~1.2 kb upstream |
| 55 | chr3:121,082,481-121,089,512 | *GSK3B* | 12 | EB360999 | nc | 7 | BE842069 | stomach | ex ~0.9 kb upstream |
| 56 | chr3:127,981,256-127,983,599 | *CHCHD6* | 8 | BF933538 | nc | 4 | DB284853 | uterus | ex ~2.2 upstream |
|  |  |  |  |  |  |  | BQ632120 | pancreas | ex ~2.2 upstream |
| 57 | chr3:128,150,281-128,152,259 | *CHCHD6* | 8 | CV408138 | nc | 6 | BI839034 | pooled: pancreas, spleen | ex ~0.5 kb upstream |
| 58 | chr3:132,994,746-133,000,019 | *CPNE4* | 20 | BF960325 | nc | 5 | AA431302 | testis | ex ~1.1 kb upstream |
| 59 | chr3:134,319,715-134,322,351 | *TMEM108* | 6 | DB338124 | nc | 2 | DB044906 | testis | ex ~1 kb upstream |
| 60 | chr3:134,613,015-134,615,651 | *BFSP2* | 7 | BM714484 | nc | 1 | BM705695 | fetal eyes | ex ~0.1 kb upstream, inclusion |
| 61 | chr3:140,834,626-140,837,629 | *NMNAT3* | 5 | BF932726 | nc | 2 | AW172383  AI002138  BF507839 etc | pooled: fetal lung, testis, B-cell  testis  colon | ex ~0.9 kb upstream  ex ~0.9 kb upstream  ex ~0.9 kb upstream, polyA |
| 62 | chr3:142,462,585-142,465,963 | *ACPL2* | 8 | DB003751 | nc | 4 | DA293825 | hippocampus | ex ~1.1 kb upstream |
| 63 | chr3:142,587,204-142,593,962 | *ZBTB38* | 8 | DR980200 | nc | 5 | AK311501 | placenta | int, within |
| 64 | chr3:155,558,412-155,563,480 | *GPR149* | 4 | BU854301 | nc | 3 | CB051105 | prostate | ex ~1.2 kb upstream |
| 65 | chr3:156,288,581-156,293,649 | *MME* | 23 | AA813982 | nc | 3 | BC106070 | adrenal cortex, carcinoma | ex ~1.4 kb upstream, polyA |
|  |  |  |  |  |  |  | BF969932 | adrenal cortex carcinoma, cell line | ex ~1.4 kb upstream |
| 66 | chr3:157,043,658-157,048,726 | *SLC33A1* | 7 | AI343815 | nc | 1 | CR741693 | kidney | ex ~1 kb upstream |
| 67 | chr3:158,085,469-158,095,607 | *LEKR1* | 14 | BU690118 | nc | 3 | BG721066 | testis | ex ~1.3 kb upstream |
| 68 | chr3:158,609,891-158,610,735 | *VEPH1* | 14 | AA417089 | nc | 6 | BX107661 | testis | ex ~30 nt upstream, inclusion |
| 69  70 | chr3:159,334,545-159,339,613  chr3:159,658,056-159,663,599 | *RSRC1*  *RSRC1* | 10  10 | BE007358  DN916985 | nc  nc | 3  7 | BU657268  DN916986 | blood  uterus | ex ~1.8 kb upstream  int ~0.3 kb upstream |
| 71 | chr3:159,669,057-159,672,435 | *RSRC1* | 10 | AW074606 | nc | 7 | AK300388 | placenta | ex ~1 kb upstream |
| 72 | chr3:159,741,594-159,747,278 | *RSRC1* | 10 | AA907607 | nc | 9 | BG209889 | HT1080 cell line | int 0.1 kb upstream |
| 73 | chr3:161,384,263-161,389,331 | *AK097161* | 10 | BM720342 | nc | 3 | AA448076 | testis | int ~2 kb upstream |
|  |  |  |  |  |  |  | AA447577 | testis | int ~2 kb upstream |
| 74 | chr3:179,928,333-179,930,867 | *AF279780* | 6 | AA834659 | nc | 5 | BG977814 | colon | ex ~0.3 kb upstream |
| 75 | chr3:179,947,765-179,951,144 | *AF279780* | 6 | DA238771 | nc | 5 | CD689536 | nasopharynx | int ~0.2 kb upstream |
| 76 | chr3:182,824,374-182,840,423 | *SOX2OT* | 5 | DB341688 | nc | 2 | DA168821 | amygdala | ex ~0.8 kb upstream |
| 77 | chr3:182,908,270-182,915,873 | *SOX2OT* | 5 | *SOX2* | c | 3 | BM474966 | small intestine adenocarcinoma | ex ~2.2 kb upstream |
| 78 | chr3:184,591,313-184,598,916 | *MCF2L2* | 30 | CK820888 | nc | 1 | BM929566 | fetal and adult eyes | ex ~3.2 kb upstream |
|  |  |  |  |  |  |  | BM683397 | fetal and adult eyes | ex ~3.2 kb upstream, polyA |
| 79 | chr3:186,906,189-186,911,257 | *IGF2BP2* | 15 | DN601766 | nc | 1 | BU660457 | blood | ex ~40 nt upstream, inclusion |
| 80 | chr3:186,841,744-186,852,513 | *IGF2BP2* | 15 | CN398357 | nc | 14 | CN398379 | embryonic stem cells | int, within |
| 81 | chr3:187,355,267-187,366,672 | *DGKG* | 25 | CK003677 | nc | 24 | AB209033 | brain | int ~4 kb upstream |
| 82 | chr13:23,669,501-23,674,500 | *SPATA13* | 15 | BX493850 | nc | 3 | DA123618 | cerebellum | ex ~1.2 kb upstream |
|  |  |  |  |  |  |  | BQ025339 | placenta | ex ~1.2 kb upstream, polyA |
| 83 | chr13:29,834,664-29,837,588 | *CR598049* | 5 | CA427697 | nc | 3 | DA977872 | synovium tissue from rheumatioid arthritis | ex ~0.4 kb upstream |
| 84 | chr13:29,810,017-29,818,794 | *CR598049* | 5 | DA917598 | nc | 4 | BQ420287 | embryonal carcinoma cell line | ex ~0.7 kb upstream |
|  |  |  |  |  |  |  | DA815089 | peripheral blood mononuclear cells | ex ~0.4 kb upstream |
| 85 | chr13:31,509,030-31,512,733 | *FRY* | 61 | U50526 | nc | 1 | AI523826 | B-cell, chronic lymphotic leukemia | ex ~0.5 kb upstream |
| 86 | chr13:32,118,279-32,125,685 | *PDS5B* | 35 | BC026240 | nc | 2 | BG546853 | lung | int, within |
| 87 | chr13:35,513,802-35,517,505 | *DCLK1* | 18 | AF019351 | nc | 3 | CA944720 | human lung epithelial cells | ex ~2 kb upstream, polyA |
| 88 | chr13:40,383,167-40,386,870 | *SUGT1L1* | 6 | CD674492 | nc | 2 | BF680244 | prostate | int, within |
| 89 | chr13:40,384,585-40,388,288 | *SUGT1L1* | 6 | AA229032 | nc | 2 | AI954659 | pooled germ cell tumors | ex ~0.2 kb upstream, inclusion |
| 90 | chr13:40,325,539-40,330,787 | *SUGT1L1* | 6 | DA223709 | nc | 3 | BI821251 | pooled brain, lung, testis | int, within |
| 91 | chr13:44,655,520-44,657,348 | *GTF2F2* | 8 | BG952866 | nc | 4 | BE784074 | retinoblastoma | ex ~0.1 kb upstream |
|  |  |  |  |  |  |  | DB199306  CN283978 | trachea  embryonic stem cell | ex ~0.1 kb upstream  ex ~0.6 kb upstream |
| 92 | chr13:47,803,211-47,806,914 | *RB1* | 27 | CV349601 | nc | 2 | BQ021681 | lung, metastatic chondrosarcoma | ex ~1.1 kb upstream, polyA |
| 93 | chr13:50,698,179-50,701,882 | *FAM124A* | 5 | AI796328 | nc | 1 | BI521123 | pooled pancreas and spleen | ex ~1.2 kb upstream |
| 94 | chr13:51,056,232-51,059,935 | *WDFY2* | 12 | BE552161 | nc | 1 | CN429964 | embryonic stem cell | ex ~0.3 kb upstream |
|  |  |  |  |  |  |  | BU177302 | melanotic melanoma | ex ~0.3 kb upstream, inclusion |
| 95 | chr13:51,223,008-51,226,367 | *WDFY2* | 12 | BF877546 | nc | 8 | AK309815 | fetal brain | int ~0.6 kb upstream |
| 96 | chr13:68,340,218-68,343,921 | BC042673 | 3 | BX479430 | nc | 1 | AA909586 | pooled: fetal lung, testis, B-cell | ex ~1.7 kb upstream |
| 97 | chr13:69,261,505-69,265,208 | *KLHL1* | 11 | DB295045 | nc | 7 | AK054609 | adrenal gland | ex, within |
| 98 | chr13:87,033,868-87,035,719 | *AF339814* | 6 | DB301073 | nc | 1 | BI522680 | pooled: 3 fetal brains | ex ~0.8 kb upstream |
|  |  |  |  |  |  |  | DA131964 | brain, alzheimer cortex | ex ~0.8 kb upstream |
| 99 | chr13:87,057,571-87,063,126 | *AF339814* | 6 | AF339815 | nc | 1 | BX107517 | pooled germ cell tumors | ex ~0.9 kb upstream |
| 100 | chr13:99,941,551-99,942,785 | *PCCA* | 24 | AL602567 | nc | 18 | AW105413 | ovary tumor | ex ~0.1 kb upstream, inclusion, polyA |
| 101 | chr13:110,591,877-110,597,432 | *ARHGEF7* | 19 | AA992958 | nc | 1 | BC043372 | brain, medulla | ex ~0.4 kb upstream, inclusion, polyA |
|  |  |  |  |  |  |  | BI829664 | brain, medulla | ex ~0.4 kb upstream |
| 102 | chr13:110,723,710-110,729,229 | *ARHGEF7* | 19 | AW867789 | nc | 11 | AK308897 | brain | int ~0.2 kb upstream |
| 103 | chr13:110,728,678-110,734,197 | *ARHGEF7* | 19 | AI040887 | nc | 14 | BX093912  CD365539  DB521911 etc | fetal liver spleen  alveolar macrophage  testis | int ~0.3 kb upstream  int ~0.1 kb upstream, polyA  int ~0.4 kb upstream |
| 104 | chr13:113,869,929-113,875,484 | *RASA3* | 24 | AA666156 | nc | 1 | DA102071 | cerebellum | ex ~1.3 kb upstream |
