# Supplementary material for: Intronic L1 Retrotransposons and Nested Genes Cause Transcriptional Interference by Inducing Intron Retention, Exonization and Cryptic Polyadenylation
Source: PLoS One. 2011 Oct 13;6(10):e26099. doi: 10.1371/journal.pone.0026099 (PMC3192792; doi:10.1371/journal.pone.0026099)
Supplement: Table S4 — Search for additional aberrant transcripts in selected host genes shown in Table S2. (DOC) [file pone.0026099.s008.doc]

Table S4. Search for additional aberrant transcripts in selected host genes shown in Table S3

| ***No*** | ***UCSC Genome Browser*** | ***Host gene*** | ***Number of exons*** | ***Location of nested gene*** | ***Nested pseudogene***  ***(?-unknown***  ***– not detected)*** | ***Location***  ***in intron*** | ***Aberrant***  ***mRNA/EST***  ***(etc >3 ESTs)*** | ***Effect (ex-exonization, int-intron***  ***retention, polyA-polyadenylation)*** |  |
| --- | --- | --- | --- | --- | --- | --- | --- | --- | --- |
|  |  |  |  |  |  |  |  |  |  |
| 1 | chr1:50,000-750,300 | AK125248 | 6 | 3 | – | – | – | – |  |
| 2 | chr1:2,241,666-2,314,336 | *MORN1* | 14 | 3, 4, 6, 10 | AluSp  **?** | 8  12 | DA061788  AA161303  CB154500  DA078666  AK310323 | int ~0.3 kb upstream  ex within  ex within  ex within  ex |  |
| 3 | chr1:3,717,668-3,764,333 | *KIAA0562* | 22 | 6, 14 | AluSc  L1MB1  L1MC5  AluSx | 12  15  20 | BC047450  CA944603  BE263389  AW249416  CA429496  BE740974  AA732726  AA715113  AA834568 etc | int ~0.4 kb upstream, polyA  int ~0.4 kb upstream, polyA  int ~0.4 kb upstream  int ~0.4 kb upstream  int ~0.6 kb upstream, polyA  int ~0.8 kb upstream  int ~0.2 kb upstream, polyA  int ~0.2 kb upstream, polyA  int ~0.2 kb upstream, polyA |  |
| 4 | chr1:6,750,099-7,760,409 | *CAMTA1* | 23 | 4 | MIR  L2  AluY/AluSx | 3  5 | BC116457  AY037153  BU594863 etc  BM800184  BG205872  CD103791 etc  AW105128 | int ~2.4 kb upstream to transcriptionally active MIR repeat, polyA  ex ~0.4 kb upstream and termination within, polyA  ex ~0.4 kb upstream and termination within, polyA  ex ~0.4 kb upstream and termination within, polyA  ex ~0.8 kb upstream to Alu repeat |  |
| 5 | chr1:9,520,674-9,570,106 | *SLC25A33* | 7 | 5 | – | – | – | – |  |
| 6 | chr1:21,638,908-21,684,403 | *NBPF3* | 15 | 2 | MIR1b  AluSx | 2  7 | DA143905  BP233319  CA428576 | ex 17 bp upstream  ex 17 bp upstream  int ~0.5 kb upstream, polyA |  |
| 7 | chr1:52,027,371-52,117,486 | *NRD1* | 33 | 2 | FLAM_C (Alu)  AluSx | 13  22 | BX348313  BX348314  BE971211  DB373343 | int ~0.7 kb upstream  int ~0.7 kb upstream  int ~0.7 kb upstream  int ~0.2 kb upstream |  |
| 8 | chr1:52,256,825-52,294,985 | *TXNDC12* | 7 | 2 | AluY | 6 | CK003983 | Int within transcriptionally active Alu repeat |  |
| 9 | chr1:71,078,096-71,300,575 | *PTGER3* | 5 | 6 | MIRb  MIRb | 3  4 | X83858  S68874  U13218 etc  X83862  EF534325 | ex ~1.7 kb upstream  ex ~1.7 kb upstream  ex ~1.7 kb upstream  ex ~0.7 kb upstream  ex ~0.7 kb upstream |  |
| 10 | chr1:117,097,745-117,115,578 | *CD2* | 5 | 4 | MER5B  MIR3 | 2  3 | DB156127  CR996162  DB122169  CT004632  BM821729 | int ~0.2 kb upstream  int ~0.5 kb upstream  int ~0.2 kb upstream  int ~0.1 kb upstream  ex 6 bp upstream |  |
| 11 | chr1:117,403,316-117,446,978 | *TTF2* | 23 | 10 | **?**  **?** | 6  15 | BQ009100  AI991587  AI652738  CV573693 | int, polyA  int, polyA  int, polyA  int |  |
| 12 | chr1:154,640,175-154,667,551 | *C1orf61* | 7 | 4, 5 | PRIMA4-int  MER5A | 2 | BC010558  BF345715  DA073766  AL535057 | ex, ~0.4 kb upstream, inclusion  ex ~0.4 kb upstream  ex ~0.4 kb upstream  int ~0.6 kb upstream |  |
| 13 | chr1:160,000,000-160,200,389 | *ATF6* | 16 | 14 | AluSx  AluSx | 2  14 | AU100274  DA766055  AW372555 | int ~0.7 kb upstream  int ~0.7 kb upstream  ex within |  |
| 14 | chr1:163,710,723-163,820,721 | *AK093132* | 6 | 4 | – | – | – | – |  |
| 15 | chr1:170,694,820-170,708,886 | *C1orf105* | 7 | 6 | L1 PA6 | 1 | AA923390 | ex ~0.2 kb upstream |  |
| 16 | chr1:178,075,179-178,113,947 | *TOR1AIP2* | 6 | 2 | AluSx  AluSp | 2 | AK057623  DB194682  BC024226 | ex ~0.9 kb upstream  ex ~0.9 kb upstream  ex within, polyA |  |
| 17 | chr1:204,581,652-204,705,463 | *SRGAP2* | 20 | 7, 17 | L1MC5 | 19 | EL593630 | ex ~1.1 kb upstream |  |
| 18 | chr1:231,529,782-231,586,088 | *KIAA1804* | 10 | 6 | – | – | – | – |  |
| 19 | chr1:232,911,529-232,922,663 | *CR596412* | 4 | 2 | – | – | – | – |  |
| 20 | chr1:239,823,127-239,870,933 | *OPN3* | 4 | 1 | – | – | – | – |  |
| 21 | chr1:241,347,095-241,493,554 | *CEP170* | 20 | 4 | AluY  **?** | 3  12 | BP229883  AA187982  BG057293 | int ~0.1 kb upstream  int  int |  |
| 22 | chr1:241,478,712-241,739,364 | *SDCCAG8* | 18 | 7, 16 | AluSq | 9 | BC032454 | ex ~1.2 kb upstream |  |
| 23 | chr1:243,975,209-244,769,875 | *SMYD3* | 12 | 5 | AluJo | 5 | BX332691 | ex within |  |
| 24 | chr1:247,166,795-247,181,891 | *PGBD2* | 3 | 2 | – | – | – | – |  |
| 25 | chr3:3,142,854-3,168,486 | *TRNT1* | 10 | 2 | L1HS  AluY | 2  4 | BU172136  AI074113  AI383100 | ex ~0.2 kb upstream  int ~0.1 kb upstream  int ~0.1 kb upstream |  |
| 26 | chr3:21,412,022-21,800,151 | *ZNF385D* | 9 | 5 | L1 PA7 | 3 | BP276600  BG194196  BG195414 etc | ex ~0.5 kb upstream  ex ~0.5 kb upstream, polyA  ex ~0.5 kb upstream, polyA |  |
| 27 | chr3:29,262,718-30,053,728 | *RBMS3* | 15 | 2, 11 | AluSg  L1 PA4 | 5  14 | BI769065  BU570213  AW779062  AF023259 etc | int ~0.7 kb upstream  int ~2.2 kb upstream  int ~2.5 kb upstream  int ~2.6 kb upstream |  |
| 28 | chr3:38,360,224-38,432,225 | *XYLB* | 19 | 2 | MIR  L1 PA3 | 9  18 | BE772384  AB015046 | int ~0.3 kb upstream  int ~4.1 kb upstream |  |
| 29 | chr3:57,710,457-57,900,461 | *SLMAP* | 21 | 5, 20 | AluSq  AluSp  AluJb  AluSp | 11  13  17  18 | AY358410  AK124200  AV727544  CR627321  DA869151 | int ~0.4 kb upstream  int within transcriptionally active Alu repeat  ex ~0.2 kb upstream to transcriptionally active Alu repeat  int ~0.6 kb upstream, polyA  int ~2.0 kb upstream |  |
| 30 | chr3:64,636,761-64,980,293 | *BC040632* | 6 | 1, 4 | MIRb  MIRb  MIR  AluJo  MIR3 | 2  3  4  5 | AL832788  AW837278  AW837450  AW837474  AW294892  DA646799  DA394131 | int ~0.1 kb upstream, polyA  int ~0.1 kb upstream  int ~0.1 kb upstream  int ~0.1 kb upstream  ex ~0.1 kb upstream, polyA  ex ~1.4 kb upstream  int ~0.6 kb upstream |  |
| 31 | chr3:65,299,621-66,000,720 | *MAGI1* | 25 | 1 | AluJb/AluSx  AluJo  MIRb | 5  6  12 | CD643150  BC062367  CR746237  AI807209 | int ~0.9 kb upstream  int ~0.7 kb upstream, polyA  int ~1.1 kb upstream  ex ~0.4 kb upstream |  |
| 32 | chr3:73,128,015-73,200,813 | *PPP4R2* | 9 | 5 | AluY  AluSx  **?** | 2  8 | BP430860  BP430901  BX089727  AI160094  DA851526 | ex within  ex within  ex within  ex within  int |  |
| 33 | chr3:78,693,464-79,800,563 | *ROBO1* | 30 | 1, 3 | AluJb  AluSx | 8  16 | DB342753  AK095256 | ex ~0.8 kb upstream  int ~0.8 kb upstream to transcriptionally active Alu repeat |  |
| 34 | chr3:100,836,531-101,002,797 | *COL8A1* | 5 | 3 | MIR3 | 2 | AF170702 | ex ~1.8 kb, inclusion |  |
| 35 | chr3:112,869,030-113,059,974 | *PLCXD2* | 5 | 3 | L1PA16 | 4 | BX090717  AI347216  AA862273 etc | ex ~1.0 kb upstream  ex ~1.0 kb upstream  ex ~1.0 kb upstream |  |
| 36 | chr3:115,520,381-116,361,813 | *ZBTB20* | 11 | 1, 5 | AluSq  L1PA16  MIRb | 5  6  7 | BX648237  BC010934  BF670367  AW237166  AA578163  BQ950787  DA120543 | int within transcriptionally active Alu repeat  ex within  ex within  ex ~0.5 kb upstream  ex ~1.0 kb upstream  ex ~1.0 kb upstream  ex ~1.0 kb upstream |  |
| 37 | chr3:116,962,540-117,674,530 | *LSAMP* | 7 | 1, 6 | HERVH | 1 | CD365478 | ex, 4 bp upstream and termination within HERV, polyA |  |
| 38 | chr3:120,097,474-120,350,873 | *IGSF11* | 9 | 3, 6 | – | – | – | – |  |
| 39 | chr3:121,021,613-121,300,052 | *GSK3B* | 12 | 7 | – | – | – | – |  |
| 40 | chr3:127,900,508-128,165,307 | *CHCHD6* | 8 | 4, 6 | – | – | – | – |  |
| 41 | chr3:132,733,683-133,500,082 | *CPNE4* | 20 | 5 | – | – | – | – |  |
| 42 | chr3:134,222,368-134,610,917 | *TMEM108* | 6 | 2 | MIR | 4 | AB051477 | ex ~0.4 kb upstream |  |
| 43 | chr3:134,601,148-134,680,517 | *BFSP2* | 7 | 1 | – | – | – | – |  |
| 44 | chr3:140,755,020-140,890,199 | *NMNAT3* | 5 | 2 | MIRb | 3 | AA917773  BX091544 | ex ~0.3 kb upstream  ex ~0.3 kb upstream |  |
| 45 | chr3:142,426,260-142,502,287 | *ACPL2* | 8 | 4 | MIRb | 3 | AW247046  AA401701  AA398161  BE265801 | ex ~1.6 kb upstream  ex ~1.6 kb upstream  ex ~0.7 kb upstream  int ~1.6 kb upstream |  |
| 46 | chr3:142,522,993-142,658,172 | *ZBTB38* | 8 | 5 | – | – | – | – |  |
| 47 | chr3:155,535,601-155,636,980 | *GPR149* | 4 | 3 | – | – | – | – |  |
| 48 | chr3:156,215,770-156,395,459 | *MME* | 23 | 3 | AluSx  AluSx | 3  20 | AK310664  DB145325  AW086484 | int within  int ~0.2 kb upstream  int ~1.2 kb upstream |  |
| 49 | chr3:157,025,917-157,055,909 | *SLC33A1* | 7 | 1 | – |  | – | – |  |
| 50 | chr3:158,020,870-158,250,039 | *LEKR1* | 14 | 3 | AluSx  L1MB7  L1MB7  MIRb  AluJb | 3  4  4  6  7 | BG494742  DB062165  DB238591  BC041967  BE568229  BX094440 etc  AI018245  BI830327 | int ~0.9 kb upstream  int ~2.0 kb upstream  int ~2.0 kb upstream  ex and termination within L1 repeat, polyA  ex within  ex within  int ~0.6 kb upstream  int ~0.2 kb upstream |  |
| 51 | chr3:158,458,213-158,711,712 | *VEPH1* | 14 | 6 | L1PA13  AluSx | 4  9 | BC078142  BC057999  BG182096 etc  DB507345 | int ~0.7 kb upstream, polyA  int ~0.7 kb upstream, polyA  int ~0.7 kb upstream  int ~1.0 kb upstream |  |
| 52 | chr3:159,300,716-159,750,615 | *RSRC1* | 10 | 3, 7, 9 | AluSx  AluSx | 5  6 | BG674990  AW801736  AW801449 | ex within  ex ~0.1 kb upstream  ex ~0.1 kb upstream |  |
| 53 | chr3:161,100,347-161,420,246 | *AK097161* | 10 | 3 | – | – | – | – |  |
| 54 | chr3:179,700,850-180,100,349 | *AF279780* | 6 | 5 | L1PA2  **?**  MIR  MIR | 3  4  3  5 | DA746847  BG185132  BU953389  BG206373 etc  BG185132  DA735220 | ex ~0.1 kb upstream  int  int  int  int ~0.7 kb upstream  ex ~2.0 kb upstream |  |
| 55 | chr3:182,761,200-182,945,319 | *SOX2OT* | 5 | 2, 3 | Charlie1 | 2 | BX091073  AA813589  AA994714 | ex ~0.1 kb upstream  ex ~0.1 kb upstream  ex ~0.1 kb upstream |  |
| 56 | chr3:184,375,095-184,633,134 | *MCF2L2* | 30 | 1, 15 | AluSx  FLAM_C  MIRb  AluJo  AluSx  AluSx  *B3GNT5**  MIRb | 3  11  15  27 | BX647790  BX642437  BX648525  AI208983  CD367113  AL704259  CN354595  AK310962  BX649003  AK124500  DA193910 | int ~0.3 kb upstream, polyA  int 17 b upstream  int ~0.6 kb, polyA  int ~0.2 kb upstream  int ~0.2 kb upstream  int ~0.3 kb upstream  int ~0.4 kb upstream  ex within Alu repeat  int ~0.2 kb upstream, polyA  ex within  ex ~1.6 kb upstream |  |
| 57 | chr3:186,832,688-187,035,757 | *IGF2BP2* | 15 | 1, 14 | AluSx  AluSx  AluJb | 3  6  8 | BG007027  BP365565  AA492507 | int ~0.3 kb upstream  ex ~0.4 kb upstream to transcriptionally active Alu repeat  int 1 b upstream |  |
| 58 | chr3:187,335,306-187,563,425 | *DGKG* | 25 | 24 | L2  MIR3 | 3  15 | BQ355323  AI911283 | int within  int ~0.4 kb upstream |  |
| 59 | chr13:23,447,001-23,780,000 | *SPATA13* | 15 | 3 | – | – | – | – |  |
| 60 | chr13:29,811,771-29,847,548 | *CR598049* | 5 | 3, 4 | – | – | – | – |  |
| 61 | chr13:31,500,360-31,770,719 | *FRY* | 61 | 1 | – | – | – | – |  |
| 62 | chr13:32,055,319-32,255,528 | *PDS5B* | 35 | 2 | AluSx  L1ME3B | 3  13 | BC070274  BU930321  AV710229  EL736543  BC039256 | int and termination within Alu repeat, polyA  int ~0.1 kb upstream  int ~0.1 kb upstream  int ~0.3 kb upstream  int and termination within L1 repeat, polyA |  |
| 63 | chr13:35,236,374-35,626,773 | *DCLK1* | 18 | 3 | AluSx | 15 | AW498483 | int within |  |
| 64 | chr13:40,253,093-40,400,332 | *SUGT1L1* | 6 | 2, 3 | MIR3  Harlequin  AluSp | 2  5 | AU311791  CN277980  DB053367  AI978959  AA961490 | ex within  ex within  ex within  int ~0.1 kb upstream  int ~0.2 kb upstream |  |
| 65 | chr13:44,583,274-44,766,173 | *GTF2F2* | 8 | 4 | – | – | – | – |  |
| 66 | chr13:47,771,727-47,960,846 | *RB1* | 27 | 2 | – | – | – | – |  |
| 67 | chr13:50,692,585-50,757,664 | *FAM124A* | 5 | 1 | – | – | – | – |  |
| 68 | chr13:51,050,676-51,242,915 | *WDFY2* | 12 | 1, 8 | AluJb | 11 | AI247854 | int ~0.4 kb upstream |  |
| 69 | chr13:68,332,193-68,359,885 | *BC042673* | 3 | 1 | – | – | – | – |  |
| 70 | chr13:69,152,237-69,592,636 | *KLHL1* | 11 | 7 | – | – | – | – |  |
| 71 | chr13:86,879,194-87,127,393 | *AF339814* | 6 | 1 | – | – | – | – |  |
| 72 | chr13:99,536,318-99,991,567 | *PCCA* | 24 | 18 | – | – | – | – |  |
| 73 | chr13:110,555,488-110,757,167 | *ARHGEF7* | 19 | 1, 11, 14 | AluY | 2 | DA072242  DB121701  DA083595 etc | int ~0.5 kb upstream  int ~0.5 kb upstream  int ~0.5 kb upstream |  |
| 74 | chr13:113,756,031-113,922,710 | *RASA3* | 24 | 1 | L1MB5 | 17 | BX492496 | int within |  |

* Protein-coding gene with 2 exons in convergent orientation
